# Anti-oncogenic activities exhibited by paracrine factors of MSCs can be mediated by modulation of *KITLG* and *DKK1* genes in glioma SCs *in vitro*

**DOI:** 10.1016/j.omto.2020.11.005

**Published:** 2020-11-26

**Authors:** Nazneen Aslam, Elham Abusharieh, Duaa Abuarqoub, Dema Ali, Dana Al-Hattab, Suha Wehaibi, Ban Al-Kurdi, Fatima Jamali, Walhan Alshaer, Hanan Jafar, Abdalla S. Awidi

**Affiliations:** 1 Cell Therapy Center, The University of Jordan, Amman 11942, Jordan; 2 Faculty of Pharmacy, The University of Jordan, Amman 11942, Jordan; 3 Department of Pharmacology and Biomedical Sciences, Faculty of Pharmacy and Medical Sciences, University of Petra, Amman 11196, Jordan; 4 Laboratory for Nanomedicine, Division of Biological & Environmental Science & Engineering (BESE), King Abdullah University of Science and Technology (KAUST), Thuwal 23955-6900, Saudi Arabia; 5 Department of Anatomy and Histology, School of Medicine, The University of Jordan, Amman 11942, Jordan; 6 Department of Medicine, School of Medicine, The University of Jordan, Amman 11942, Jordan; 7 Department of Hematology and Oncology, Jordan University Hospital, The University of Jordan, Amman 11942, Jordan

**Keywords:** mesenchymal stem cells, glioma stem cells, gliospheres, conditioned media, WJ-MSCs, BM-MSCs, microarray, signaling pathways, CSC arrays, stemness

## Abstract

Cancer stem cells (CSCs) use their stemness properties to perpetuate their lineage and survive chemotherapy. Currently cell-based and cell-free therapies are under investigation to develop novel anti-cancer treatment modalities. We designed this study to investigate how cell extracts of mesenchymal stem cells affect the growth of glioma stem cells *in vitro*. Gliospheres were generated from the U87MG cell line and treated with conditioned media of Wharton’s jelly and bone marrow mesenchymal stem cells. The effects were investigated at the functional and molecular levels. Our results showed that conditioned media from both types of mesenchymal stem cells changed the morphology of spheres and inhibited the proliferation, invasion, and self-renewal ability of glioma stem cells. At the molecular level, metabolism interruption at oxidative phosphorylation, cell cycle arrest, cell differentiation, and upregulation of the immune response were observed. Furthermore, this effect was mediated by the upregulation of the *DKK1* gene inhibiting the Wnt pathway mediated by growth factor activity and downregulation of the *KITLG* gene activated by growth factor and cytokine activity, inhibiting multiple pathways. We conclude that different types of mesenchymal stem cells possess antitumor properties and their paracrine factors, in combination with anti-immune modalities, can provide practical therapeutic targets for glioblastoma treatment.

## Graphical Abstract

**Figure:**
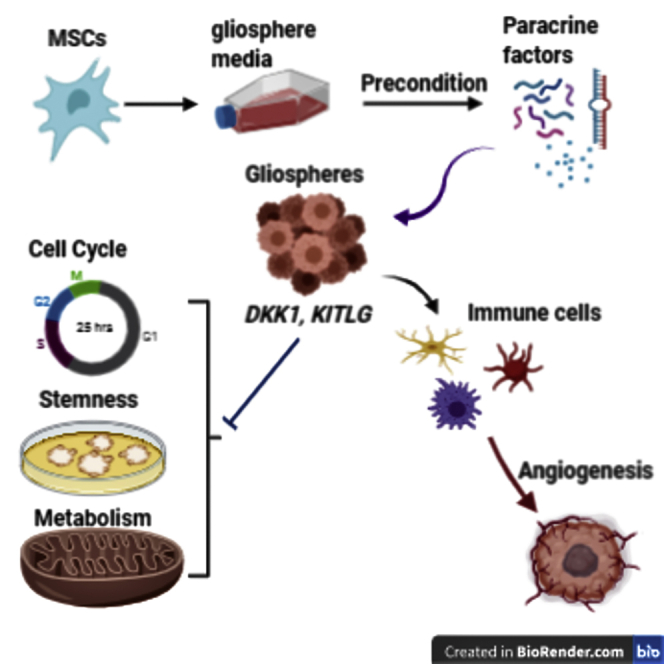

## Teaser

Glioblastoma has limited treatment options due to the selective blood-brain barrier. Paracrine factors from mesenchymal stem cells can cross this barrier and exhibited anti-oncogenic potential for glioma stem cells *in vitro*. These soluble factors can be a tool as a combinatorial therapy for the treatment of glioblastomas.

## Introduction

The modern theory of carcinogenesis focuses on the presence of malignant transformations in adult tissue stem cells.^1^ The theory of cancer stem cells (CSCs) is old, as it was first described in 1973.^2^ Later, in 1997, the existence of a heterogeneous tumor cell population was mentioned in leukemia for the first time.^3^ Analysis of this cell population revealed stem cell properties, such as self-renewal capacity, high proliferation rate, and maintenance of the tumor cell population.^4^^,^^5^ These properties form the basis of the modern accepted hypothesis of CSCs.^6^, ^7^, ^8^

The CSC hypothesis gained credibility because all main cancer-origin theories (genetic/epigenetic events and chemical-, infection-, and virus-induced carcinogenesis) indicated that the tissue stem cell is involved in the generation of cancer.^3^^,^^9^^,^^10^ Moreover, recent studies also suggest that CSCs are a major driving force for tumorigenesis, metastasis, aggressiveness, and resistance to treatment.^11^ The presence of CSCs has been reported in both hematologic malignancies and solid tumors (i.e., breast cancer, brain tumors, malignant melanoma, or prostate cancer).^12^^,^^13^

Glioblastoma multiforme (GBM) is the most malignant type of brain tumor and is still incurable, with the overall survival rate being less than 15 months.^14^, ^15^, ^16^ The highly infiltrative growth of GBM and its resistance to chemotherapies/radiotherapies prevent complete elimination of tumor cells, despite improvements made in surgical techniques and therapeutic protocols.^17^^,^^18^ The highly tumorigenic subpopulation of glioblastoma CSCs (GSCs) is thought to be one of the reasons for a high GBM recurrence rate.^19^ The resistance to radiation and chemotherapy of GSCs suggests that new therapeutic approaches are needed to focus on specific targeting of GSCs to improve the survival of GBM patients. Many current therapies, such as the drugs that target different signaling pathways, tumor transcription factors, cells in the tumor microenvironment, and the use of tumor-inhibiting microRNAs (miRNAs),^20^ are presently being investigated in the development of novel therapeutic targets.

However, targeting GSCs can be tricky, as GSCs would normally be quiescent and enter the cell cycle only after exposure to external stimuli such as growth factors.^21^ Therefore, GSCs would only be vulnerable to treatments while they are in an actively growing state.

Cellular therapy for cancer is being revisited because of using mesenchymal stem cells (MSCs) for cancer treatment. MSCs are multipotent stem cells most often isolated from perinatal sources such as umbilical cord Wharton’s jelly (WJ-MSCs), placenta (PL-MSCs), and adult tissues such as bone marrow (BM-MSCs) and adipose tissue (AT-MSCs).^22^ They have been shown to have wide therapeutic potential because of their immunomodulatory ability, wound- and neoplasm-directed homing, and tissue repair ability.^23^^,^^24^ Additionally, MSCs have been shown to cross the blood-brain barrier (BBB), a characteristic that represents an important aspect when considering MSCs as a therapeutic option for brain tumors.^25^ Recently, pre-clinical studies demonstrated that MSCs might suppress the growth of GBM cells, although conflicting studies have also found the opposite effect.^26^ However, the mechanisms by which MSCs may suppress GBM growth have not been illustrated yet.

There has been active research to enhance the anticancer effects of MSCs, such as overexpressing anticancer genes^27^ or using engineered delivery systems with an anticancer drug.^28^ However, these methods can reduce the viability of MSCs, modify endogenous genes, or exert toxicity on normal cells. Such negative effects limit the clinical application of cell-based therapy. Moreover, the issue of whether the MSCs of the tumor microenvironment and their molecular crosstalk with other cells results in tumor-suppressive effects or favors tumor promotion is still unclear.^29^

It has been reported that paracrine factors of MSCs showed enhanced beneficial effects on recovery from injury or disease in some experimental models.^30^, ^31^, ^32^, ^33^, ^34^, ^35^ MSCs harvested from numerous anatomical locations display similar immunophenotypic profiles. However, the secretome of MSCs appears to vary significantly, depending on the age of the host and diverse stimuli present in the niches where the cells reside.^36^ To improve the therapeutic ability of MSCs, the composition of the secretome of MSCs can be modulated by preconditioning the MSCs during *in vitro* culture.^37^ Preconditioning of MSCs by hypoxia, inflammatory stimulus, and other factors/conditions before their use in therapy is a new strategy currently being investigated.^38^

Based on this knowledge, we sought to investigate the effects of the conditioned media (CM) from two different types of MSCs preconditioned with a GSC microenvironment to explore the same biological mechanisms and signaling pathways that are associated with anti- or pro-tumorigenic effects *in vitro*. In doing so, we evaluated the effects of factors secreted (cytokines, chemokines, miRNAs, or growth factors in CM), under the same culture conditions, from two kinds of MSCs, BM-MSCs (BM1, BM2, BM3) and WJ-MSCs (WJ1, WJ2, WJ3), on GSC proliferation, invasion, and self-renewal capability. Furthermore, the genetic changes at signaling pathway levels were explored by microarray analysis followed by validation of the effect on the pluripotency of GSCs with human CSC arrays *in vitro*.

## Results

### Preliminary data

Figure S1 shows the results of preliminary experiments for different concentrations of CM (50% and 100%) on the CD133^+^ population sorted from the U87MG cell line. It was noted that cells treated with different concentrations of CM changed their morphology at 48 and 72 h and started to become linear rather than making spheres (Figure S1A). Similarly, we found that gliosphere CM (GSCM) harvested from BM1-MSCs showed statistically significant inhibition of proliferation of GSCs in any formulation of CM concerning both controls at 96 h (p < 0.01, p < 0.0001). However, CM from WJ3-MSCs showed significant inhibition from 50% concentration of serum-free CM (SFCM) + gliosphere media (GSM) only (p < 0.05) (Figure S1B). This prompted us to stretch the experiment for the usual passage time of 7 days. It was also noted that GSCs kept on growing even in serum-free conditions (Dulbecco’s modified Eagle’s medium [DMEM]-F12 only) without the addition of growth factors and supplements under ultralow attachment culture conditions (3D) (Figures S1A and S1B). This showed the plasticity of GSCs, modifying themselves according to the change in the environment.^41^ However, to determine the effect of CM from MSCs specifically for GSCs in the 3D culture system under normal growth conditions, we no longer used the serum-free media (SFM) as a control for further assays. Based on the preliminary data of morphology and proliferation, 100% CM (GSCM) was selected to perform the further experiments in a ratio of 1:9 with fresh GSM (10% GSM/90% CM) to replenish the growth factors.

### Enrichment and characterization of CD133^+^ population

Figure S2A depicts the flow cytometric evaluation of the CD133^+^ population present in the U87MG cell line (P3–P7) before sorting and enrichment of a positive population after sorting. Before cell sorting, we found that the average percentage of the CD133^+^ population was 5.1% in the cell line. After sorting, the CD133^+^ population was enriched to 48% while the negative population still showed 2.1% of CD133^+^ cells (Table 2). To determine whether successive cultures of sorted cells have affected the enrichment of the CD133^+^ population in CSC media (GSM), we also determined the enrichment of the sorted positive population at a random passage (G5), which was above 90% with almost uniform sphere morphology (Figure S2B). The enriched glioma stem cell line with a 90% CD133^+^ population was used for all of the experiments.

**Table 1:** Primary and secondary antibodies used for immunocytochemistry

Antibodies	Catalog no.	Manufacturer
Primary antibody		
Anti-CD133	Ab19898	Abcam (UK)
Rabbit recombinant monoclonal mushashi1/Msi1	ab199781	Abcam (UK)
GFAP monoclonal antibody (ASTRO6)	MA512023	Invitrogen
OLIG1/OLIG2/OLIG3 monoclonal antibody (257224)	MA523805	Invitrogen
Beta-3 tubulin monoclonal antibody (2 28 33)	322600	Invitrogen
Nestin monoclonal antibody (10C2)	14-9843-82	Thermo Fisher Scientific
Sox 2 antibody	48-1400	Thermo Fisher Scientific
Anti-PAX6 antibody	ab5790	Abcam (UK)
Anti-Sox1 antibody	ab87775	Abcam (UK)
Secondary antibody		
Goat anti-mouse immunoglobulin G (IgG)1-Alexa Fluor 488	A21121	Thermo Fisher Scientific
Goat anti-rabbit IgG (H+L)-Alexa Fluor 546	A11035	Thermo Fisher Scientific
Goat anti-mouse IgG (H+L)-Alexa Fluor 546	A11003	Thermo Fisher Scientific

**Table 2:** Percentage of CD133 population before and after sorting with mean ± SD

	CD133 before sorting (%)	After sorting +Ve population (%)	After sorting −Ve population (%)
1	4.17	49.75	0.62
2	7.24	45.02	4.85
3	5.4	40.45	0.65
4	3.7	56.3	2.3
Mean ± SD	5.127 ± 1.58	47.88 ± 6.77	2.105 ± 1.99

SD, standard deviation.

### Morphological characteristics of sorted populations

**Figure 1:**
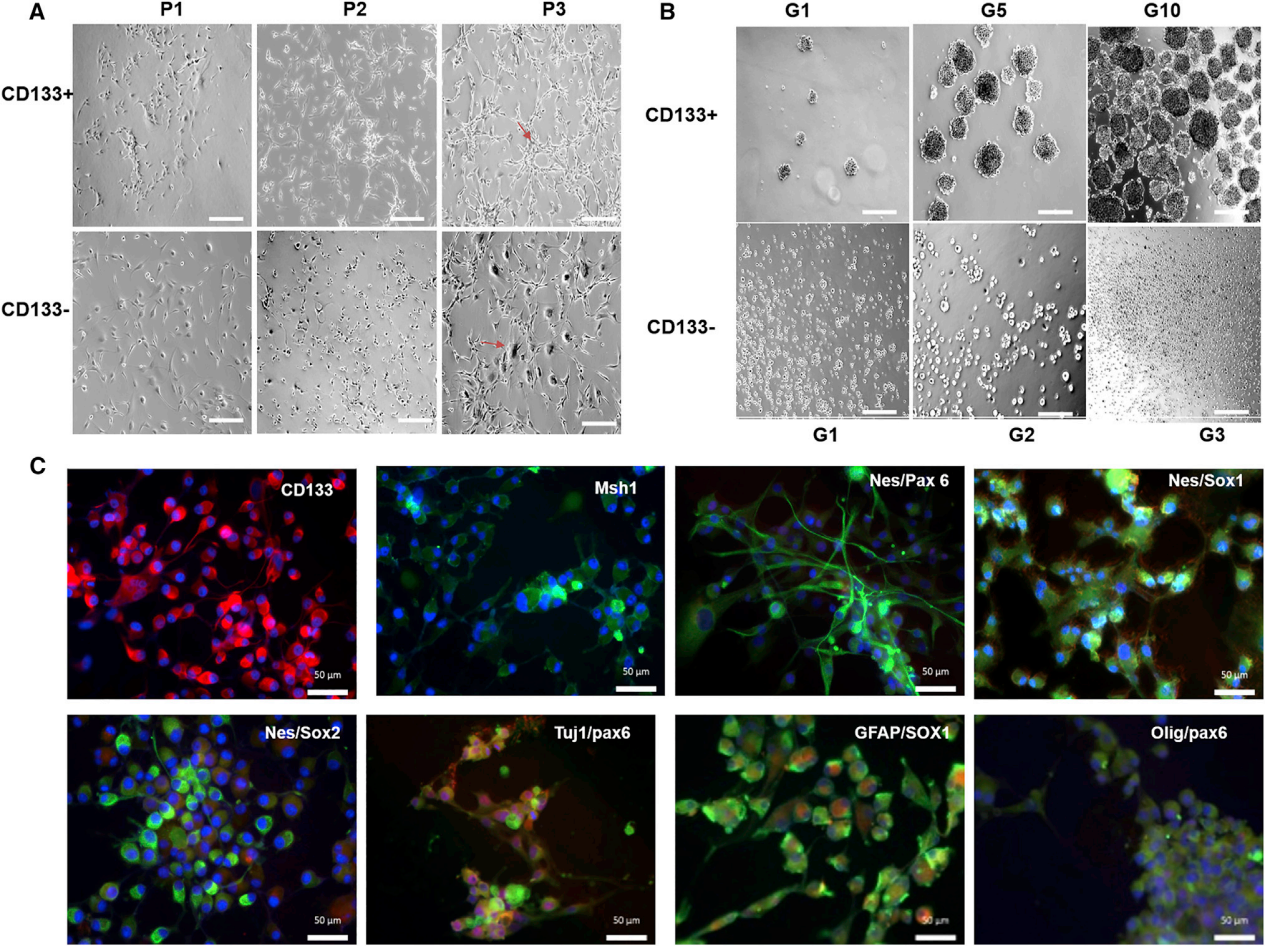
Characterization of sorted population from U87MG cells based on self-renewal and immunocytochemical assay (A) Morphological evaluation of sorted CD133^+^ and CD133^−^ populations in adherent culture from passages 1–3 (P1–P3). Scale bars, 100 μm. Arrow shows the mesh network and stellar morphology of U87MG cells in the CD133^+^ population at P3. The CD133^−^ population showed large cells indicating the presence of differentiated progeny at P3 (arrow). (B) Morphological evaluation of sorted CD133^+^ and CD133^−^ populations in sphere culture (3D). CD133^+^ population showed self-renewal ability by forming spheres until passage 10 (G10). However, the CD133^−^ population stops making spheres at the third passage (G3). Scale bars, 100 μm. (C) Immunocytochemistry of sorted CD133^+^ population for GSC (CD133 and Msh1), neuronal lineage (nestin, sox1, sox2, and pax6), and differentiated (GFAP, Tuj1, and Olig123) markers in combinations (merged) at G13. Scale bars, 50 μm. See Figure S7.

Sorted populations were divided into positive and negative fractions and were cultured in adherent (2D) and sphere (3D) systems. In adherent cultures, the CD133^+^ population made an interconnected mesh network from passage 1, and at passage 3, cells showed a conspicuous stellar morphology as for U87MG cells (Figure 1A, P3, arrow), whereas the CD133^−^ population showed larger cells indicating the presence of a differentiated progeny (Figure 1A, P3, arrow). Moreover, the negative population showed a slight stellar morphology at later passages (P5–P6), which might be due to the presence of residual CD133^+^ cells in the negative fraction or some CD133^−^ cells may have acquired the state of CD133^+^ cells.^42^

Similarly, in spheroid culture, a positive fraction started making spheres from G1 and continued until G12. However, the negative fraction did not make spheres after G3, and at later passages, cells tend to be adherent rather than spheroids (same as in Figure S1A). This negative population failed to form spheres and was kept as a negative control for molecular analysis.

### Immunocytochemistry of gliospheres

Figure 1C shows three panels of markers selected for the characterization of the glioma stem cells in actively growing spheres (gliospheres). The markers were glioma stem cell markers (CD133 and Msh1), neuronal lineage markers (nestin, sox1, sox2, and pax6), and differentiated markers (GFAP, Tuj1, and Olig123). Because the glioma spheroids contain heterogeneous populations^43^ comprised of the stem and differentiated cells, we found the substantial presence of all of the selected markers in merged images (see Figure S7).

### CM inhibited sphere formation, proliferation, invasion, and clonogenicity of GSCs

**Figure 2:**
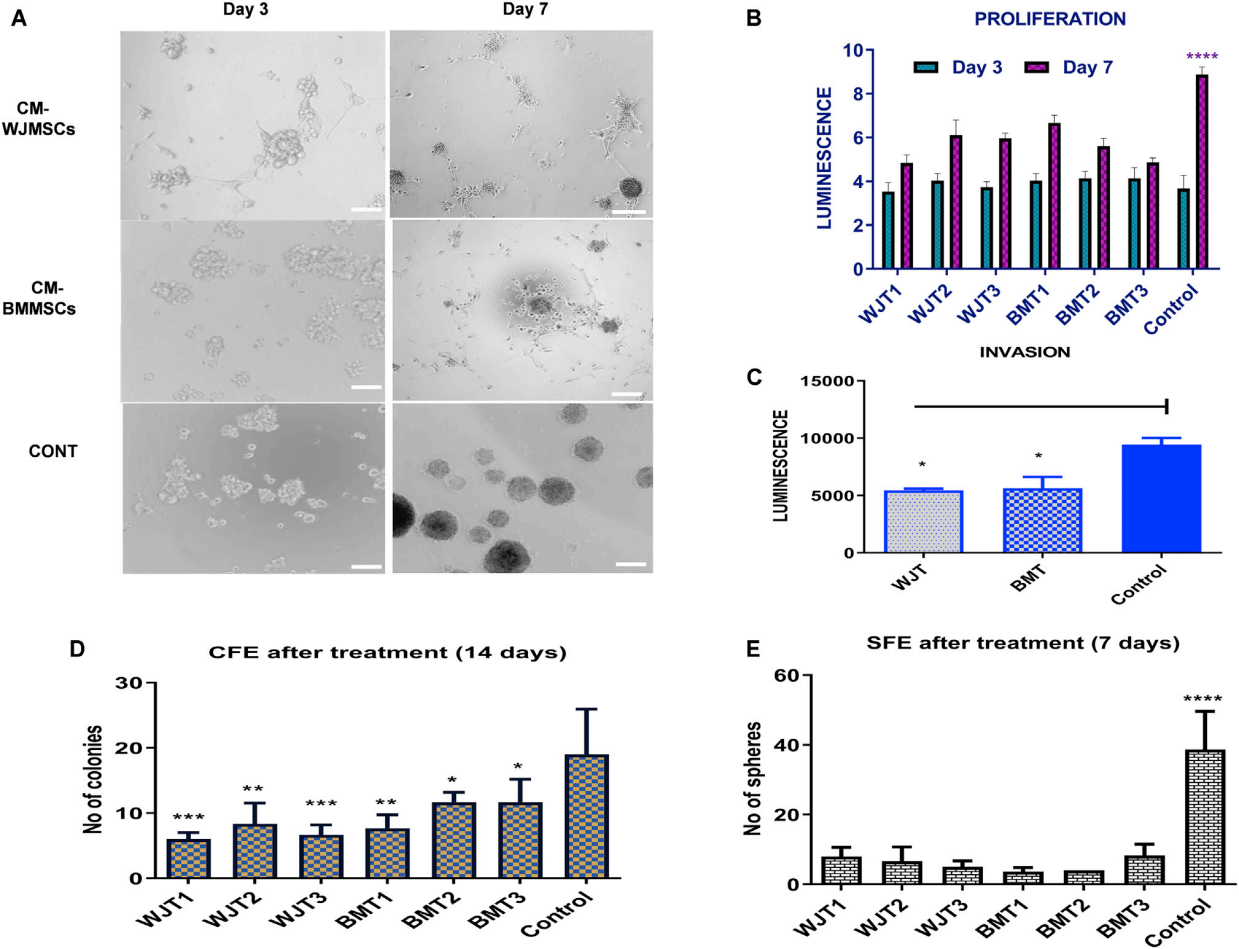
Effect of CM from MSCs on GSC (gliospheres) morphology, proliferation, invasion, and clonogenicity (A) Representative images of the morphological changes occurring in gliospheres with CM at days 3 and 7 as compared to control. Scale bars, 100 μm. (B) Effect of CM from two types of MSCs on the proliferation of gliospheres at days 3 and 7. The proliferative ability of GSCs was significantly inhibited at day 7 (p < 0.0001) in comparison to control. (C) Effect of CM from two types of MSCs on the invasive ability of gliospheres at day 7. Both treatments significantly inhibited the invasive potential of GSCs (p < 0.05). (D) Self-renewal ability of treated glioma stem cells in adherent culture (CFE) as significantly reduced at day 14 (p < 0.05, p < 0.01, and p < 0.001). (E) Self-renewal ability of treated glioma stem cells in sphere culture (SFE) was significantly reduced at day 7 (p < 0.0001).

Gliospheres were treated with CM from two types of MSCs for 7 days and the results are shown in Figure 2. Morphologically, spheroids tend to change their form as early as on day 3 (D3) where cells started to become linear rather than spheroids. At D7, cells started to branch out as for adherent culture, despite growing in ultralow stem cell environment (Figure 2A). Concerning this, a 3D Glo viability assay showed that cells’ proliferative ability was significantly decreased at D7 (p < 0.0001) as compared to control (Figure 2B) and the effect was same from all individual biological samples. Additionally, CM from both types of MSCs (averages) significantly reduced the invasive ability of GSCs (p < 0.05) (Figure 2C) As a fraction of total, the reduction in invasion was 27% (WJ) and 26% (BM) as compared to 45% from control (Figure S1C).

To investigate whether the effect of CM from both types of MSCs is irreversible, a clonogenic assay was performed. It was noted that the colony-forming efficiency of GSCs was significantly reduced both adherently and in spheroids compared to the control (p < 0.05, Figures 2D and 2E). It was found that the average sphere/colony formation from control was 39/19, while the reduction in sphere/colony formation was 5/11 from BM-MSCs and 7/6 from WJ-MSCs (Figure S1D).

### Molecular analysis

#### CM inhibited the metabolism and cell cycle while activating the immune response at the molecular level (microarray)

**Figure 3:**
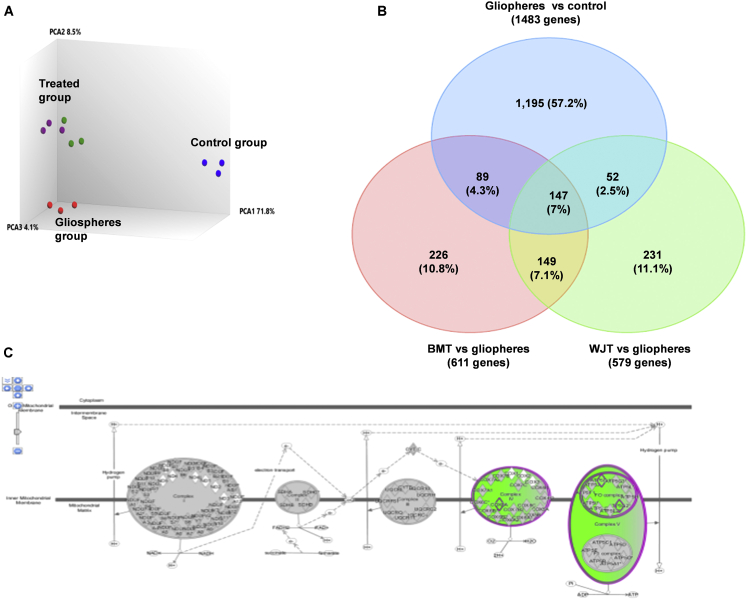
Microarray results of gliospheres and treated gliospheres using TAC analysis and IPA analysis (A) PCA showing the distribution of clusters of control and treated gliospheres. Blue indicates the negative control group, red indicates the gliosphere group, and purple and green indicate the treated group. Treated groups are clustered together at the same position close to gliospheres while the control group has its unique ancestor. (B) Venn diagram showing the distribution of DEGs among different groups. (C) Dysregulation of the oxidative phosphorylation pathway in treated gliospheres by IPA analysis. The schematic illustrates complexes I–V of the mitochondrial electron transport chain (green indicates downregulated complexes V–VI).

Principal-component analysis (PCA) demonstrating global gene-expression changes among different treatments (BMT versus gliospheres, WJT versus gliospheres, gliospheres, and −VeS) and any outliers (Figure 3A) shows that each type of group clustered together with a clear separation between them, and no outlier was observed. Both BMT and WJT gliospheres are closer to the gliospheres (+VeS) while the –Ve control (−VeS) has its unique ancestor.

To generate a list of differentially expressed genes (DEGs) between different types of treatments in comparison to controls, the eBayes ANOVA method was used, and a filtering criterion of fold change (FC) −2 or less or ≥2 and a p value ≤0.05 were applied. The detailed description of DEGs has been provided in the supplementary file (Excel 1) and shown in the Venn diagram (Figure 3B).

##### Gliospheres

For 1,483 DEGs between gliospheres and the negative control (Figure 3C), 81 significant canonical pathways were identified by Ingenuity Pathway Analysis (IPA) software (QIAGEN, USA). Based on the *Z* score evaluation, supremely activated pathways included cholesterol biosynthesis, mevalonate pathway 1, systemic lupus erythematosus in the T cell signaling pathway, and Toll-like receptor signaling while *PD-1*/*PD-L1* cancer immunotherapy pathway was inhibited (Table 3).

**Table 3:** Significantly enriched canonical pathways (IPA)

Top canonical pathways based on significance	−log (p value)	*Z* score
Gliospheres versus control (cell line and −e population)		
Superpathway of cholesterol biosynthesis	15	activated
Cholesterol biosynthesis I	11	inhibited
Cholesterol biosynthesis II (via 24,25-dihydrolanosterol)	11	activated
Cholesterol biosynthesis III (via desmosterol)	11	inhibited
Systemic lupus erythematosus T cell signaling pathway	4.5	activated
Superpathway of geranylgeranyl diphosphate biosynthesis I (via mevalonate)	4.5	activated
Mevalonate pathway I	4	activated
Toll-like receptors signaling	3.5	activated
Sperm motility	2	activated
Dendritic cell maturation	2	activated
Cardiac hypertrophy signaling	2	activated
Calcium-induced T lymphocyte apoptosis	2	activated
PD-1/PD-L1 cancer immunotherapy	2	inhibited
eNOS signaling	1.5	activated
Stearate biosynthesis I (animals)	1.5	activated
WJT versus gliospheres		
TREM1 signaling	2.65	activated
G6P signaling pathway	1.75	activated
NF-κB activation by viruses	1.65	activated
Oxidative phosphorylation	1.5	inhibited
BMT versus gliospheres		
PD-1/PD-L1 cancer immunotherapy	2.5	activated
Calcium-induced T lymphocyte apoptosis	2.1	inhibited
Oxidative phosphorylation	1.35	inhibited

##### WJT gliospheres

**Figure 4:**
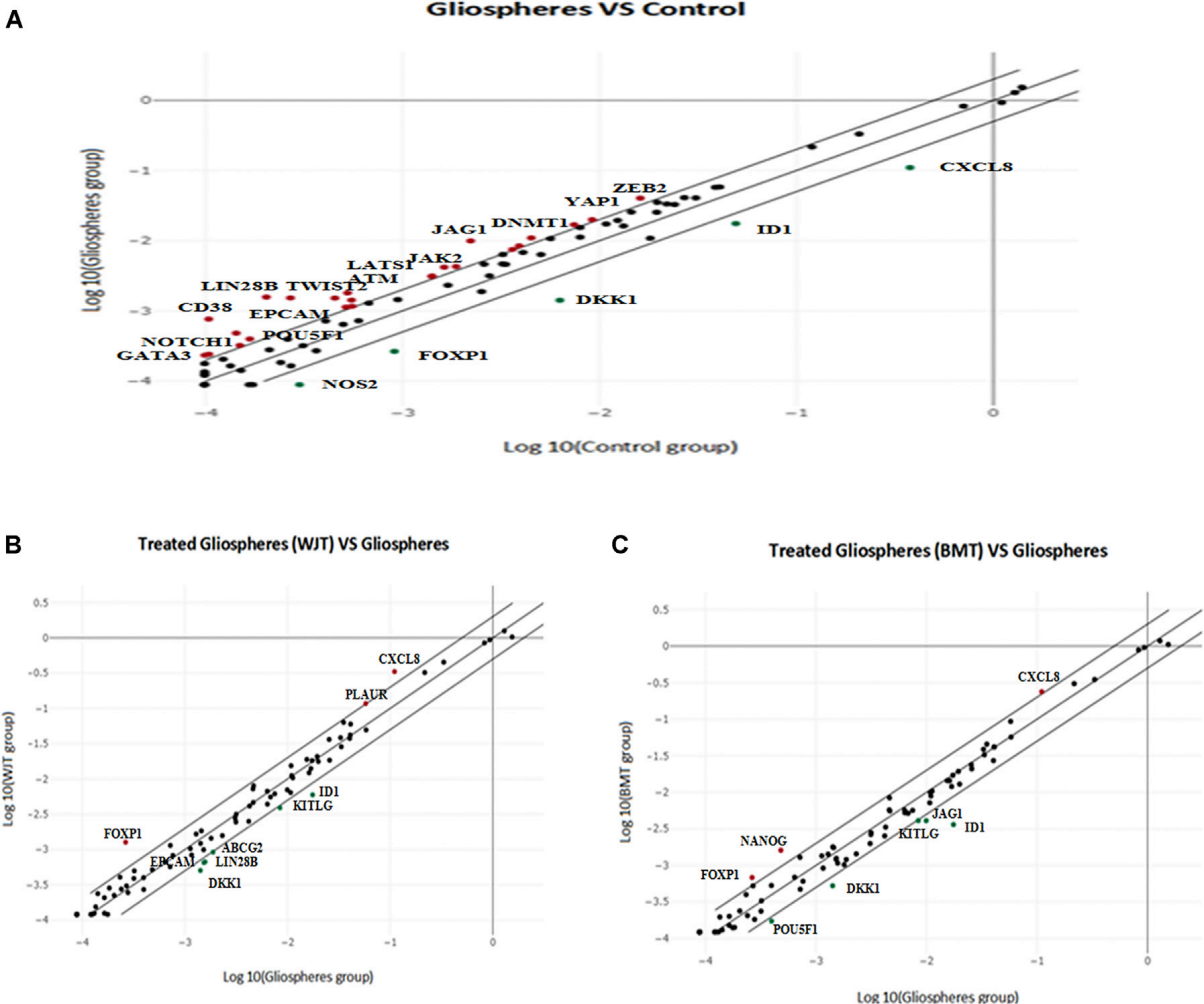
Scatterplots of DEGs in gliospheres and treated gliospheres using a human cancer stem cell (CSC) array CSCs were determined with the RT2 Profiler PCR array (catalog no. PAHS-176Z, QIAGEN, USA). The upregulated genes are marked with red points, while the downregulated genes are marked with green points, and the unchanged genes are marked with black points. (A) Gliospheres comprised of glioma stem cells sorted from U87MGMG cells (ATCC HTB14) compared to control (cell line and –ve population). (B) Treated gliospheres with conditioned media of Wharton’s jelly mesenchymal stem cells (WJT) in comparison to gliospheres. (C) Treated gliospheres with conditioned media of bone marrow mesenchymal stem cells (BMT) in comparison to gliospheres.

For 579 DEGs between WJT and gliospheres applied to IPA (Figure 4B), 57 canonical pathways were identified as significant. These include *TREM1* signaling, *GP6* signaling, and nuclear factor κB (*NF*-*κB*) activation by virus as activated, while oxidative phosphorylation was inhibited (Table 3).

##### BMT gliospheres

For 611 DEGs between BMT and gliospheres applied to IPA (Figure 4B), 36 significant canonical pathways were identified. Based on the *Z* score, the topmost pathways include *PD-1/PD-L1* cancer immunotherapy (activated), calcium-induced T lymphocyte apoptosis, and oxidative phosphorylation (inhibited) (Table 3).

It was noted that both treatments commonly inhibited oxidative phosphorylation at complexes IV–V (green, downregulated, Figure 3C).

#### Gene Ontology (GO) and pathway analysis (microarrays)

To investigate the functional importance and biological processes associated with the differentially expressed signature genes with each treatment (CM from BM and WJ MSCs) as well as in gliospheres (+VeS versus –Ve), GO analysis was performed using the open-source DAVID gene annotation website (https://david.abcc.ncifcrf.gov/) and NetworkAnalyst (3.0). The signature upregulated and downregulated genes were analyzed separately. Based on the significance (p ≤ 0.05 and hits), the top Kyoto Encyclopedia of Genes and Genomes (KEGG) pathways and biological processes for each type of treatment group are shown in Figures S3–S5.

##### Gliospheres

For upregulated genes in gliospheres, ribosome biogenesis in eukaryotes, steroid biosynthesis, antigen processing and presentation, asthma, and terpenoid backbone biosynthesis were identified as significantly upregulated KEGG pathways. The downregulated genes were involved in the *HIF-1* signaling pathway, protein export, protein processing in the endoplasmic reticulum, ferroptosis, glycosphingolipid biosynthesis ganglio series), and autophagy (Figure S3).

Similarly, upregulated genes were represented by top biological processes of lipid, steroid, and cholesterol metabolic processes, antigen processing and presentation, fatty acid biosynthesis and the metabolic process, and immune response, while the downregulated genes were represented by top biological processes of negative regulation of the apoptotic process, cell death, angiogenesis, cell adhesion, the rhythmic process, and synaptic vesicle exocytosis (Figure S3).

##### BMT and WJT gliospheres

From both types of treatment (CM of BM-MSCs and WJ-MSCs), we found almost the same significantly upregulated KEGG pathways such as ECM-receptor interaction, the *AGE-RAGE* signaling pathway in diabetic complications, focal adhesion, protein digestion and absorption, the phosphatidylinositol 3-kinase (PI3K)*-Akt* signaling pathway, and proteoglycans in cancers, while ribosome biogenesis in eukaryotes, systemic lupus erythematosus, asthma, and antigen processing and presentation were significantly downregulated or inhibited (Figures S4 and S5)

In the same manner, GO terms for upregulated biological processes were enriched in cell adhesion, angiogenesis, receptor-mediate endocytosis, heart and skeletal system development, cellular defense response, cell differentiation, and blood coagulation, while the downregulated genes were represented by top biological processes of DNA replication, regulation of cell cycle, RNA splicing, DNA repair, cell cycle, antigen processing and presentation, RNA splicing, and protein transport (Figures S4 and S5).

Based on pathway analysis and biological processes, it was noted that CM from both types of MSCs inhibited metabolism, arrested the cell cycle, and activated the immune response in GSCs.

### RT2 Profiler PCR array

#### CM inhibited cell proliferation and the pluripotency of glioma stem cells and induced cell differentiation and the immune response

To validate the findings of microarray analysis, a specific human CSC array was used to investigate whether the cell cycle was arrested and multipotency or pluripotency of glioma stem cells was affected and which specific genes and pathways are involved in causing this effect. The results of the CSC array for gliospheres are summarized in (Table 4). After normalization, out of 84 genes of the arrays, 29 genes were identified as DEGs (24 upregulated, 5 downregulated) in gliospheres. A scatterplot for gliospheres (gliospheres versus control, Figure 4A) shows the distribution of gene expression changes along the central diagonal line. Significantly upregulated genes (p < 0.05) were *DNMT1*, *GSK3B*, *IKBKB*, *ITGA6*, *LATS1*, *LIN28B*, *WWC1*, *ZEB2*, and *YAP1*, while the genes observed with higher fold regulation were *LIN28B*, *JAG1*, *EPCAM*, *TWIST2*, *ATM*, *NANOG*, and *CD38* (Table 4). However, one gene was found significantly upregulated (p < 0.05) and with high fold regulation *LIN28B* (7.72).

**Table 4:** Upregulated and downregulated genes in gliospheres comprised of glioma stem cells sorted from cell line using human cancer stem cell array compared to control (cell line and –Ve population)

Gene symbol	Fold regulation	p value
ATM	3.42	0.078675
CD38	7.36	0.050531
DNMT1	2.43	0.046934
EGF	2.58	0.264680
EPCAM	5.66	0.174573
GATA3	2.31	0.249860
GSK3B	2.27	0.026471
IKBKB	2.07	0.041685
ITGA6	2.19	0.016071
JAG1	4.48	0.126777
JAK2	2.57	0.232893
KITLG	2.16	0.115002
LATS1	2.22	0.033813
LIN28B	7.72	0.031777
MAML1	2.20	0.088189
NANOG	3.36	0.134051
NOTCH1	2.15	0.141905
POU5F1	2.36	0.252004
PROM1	2.35	0.298908
TAZ	2.28	0.071685
TWIST2	3.35	0.142327
WWC1	2.09	0.030530
YAP1	2.17	0.032586
ZEB2	2.50	0.000967
DKK1	−4.45	0.063695
FOXP1	−3.44	0.156756
ID1	−2.81	0.104330
CXCL8	−3.43	0.483770
NOS2	−3.39	0.275178

Cancer stem cells were determined with the RT^2^ Profiler PCR array (catalog no. PAHS-176Z, QIAGEN, USA).

Scatterplots for WJT and BMT gliospheres have shown the same pattern as depicted in Figures 4B and 4C and Table 5. Based on fold regulation, it was noted that both treatments downregulated most of the genes that were upregulated in gliospheres. We found almost the same upregulated genes by both treatments such as *CXCL8* and *FOXP1*, while *PLAUR* was upregulated by WJT, and *NANOG* remained unchanged by BMT. However, we found some variations in downregulated genes by both treatments. Common genes significantly (p < 0.05) downregulated by both treatments were *DKK1* and *KITLG*. Since *DKK1* was already downregulated (FC less than −4.45) in gliospheres too, only one gene was significantly (p < 0.05) downregulated by both treatments, which is *KITLG*, while *DKK1* seemed to be significantly upregulated (FC less than −2.80 and −2.69) as compared to gliosphere (Table 4).

**Table 5:** Upregulated and downregulated differentially expressed genes in treated gliospheres with conditioned media of WJ-MSCs (WJT) and BM-MSCs (BMT) in comparison to gliospheres (control) using a human cancer stem cell array

Gene symbol	WJ treated		BM treated	
	Fold regulation	p value	Fold regulation	p value
FOXP1	4.80	0.070962	2.58	0.096343
CXCL8	3.02	0.678317	2.16	0.832641
PLAUR	2.03	0.228839	–	
NANOG	–	–	3.35	0.106006
ABCG2	−2.05	0.066120	–	
DKK1	−2.80	0.032991	−2.69	0.045981
ID1	−2.94	0.088560	−4.85	0.056106
KITLG	−2.16	0.038884	−2.06	0.040332
EPCAM	−2.34	0.143145	–	–
LIN28B	−2.35	0.083791	–	–
JAG1	–	–	−2.43	0.199360
POU5F1	–	–	−2.29	0.180843

Cancer stem cells were determined with the RT^2^ Profiler PCR array (catalog no. PAHS-176Z, QIAGEN, USA).

Uniquely downregulated genes based on fold regulation by WJT were *ABCG2*, *EPCAM*, and *LIN28B*, while uniquely downregulated genes by BMT were *JAG1* and *POU5F1* (Table 5).

#### Pathway analysis and GO terms of CSC arrays

##### Gliospheres

**Figure 5:**
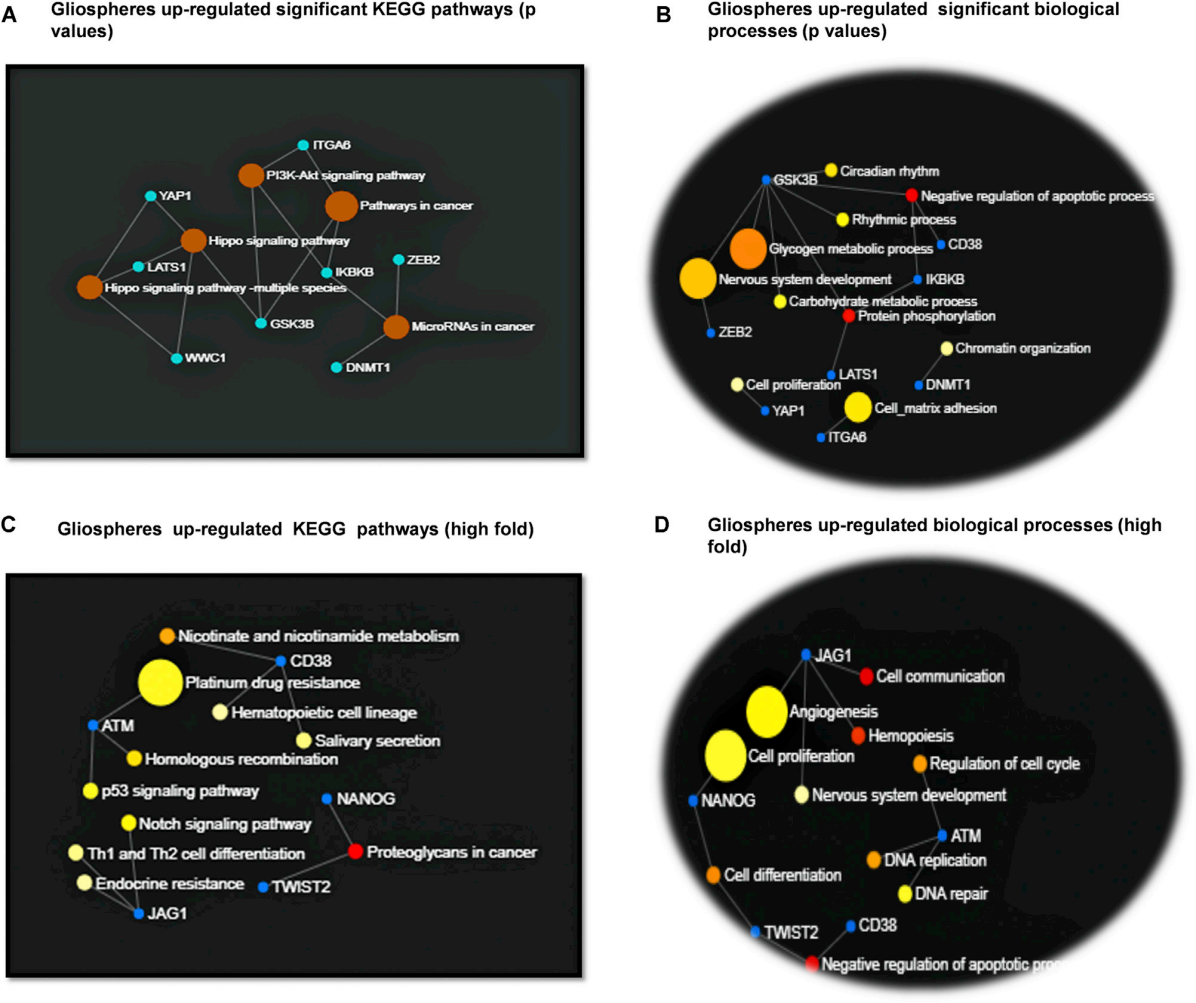
Gene ontology terms of gliospheres from human CSC arrays (A and C) Activated KEGG pathways according to (A) significant genes (p values) and (C) high fold regulation in gliospheres. (B and D) Upregulated biological processes according to (B) significant genes (p values) and (D) high fold regulation in gliospheres. Color variations shows the significance level, with red being the highest (p < 0.0001) and white the lowest (p < 0.05).

Figure 5 shows the activated and inhibited pathways and biological processes of significantly upregulated DEGs in gliospheres. It was noted that the most significant activated pathways in gliospheres were *Hippo* signaling, *P13/Akt* signaling, pathways in cancer, and microRNAs in cancer (Figure 5A). With this, highly significant biological processes (based on p values) in gliospheres were protein phosphorylation, negative regulation of apoptotic processes, glycogen and carbohydrate metabolic processes, cell proliferation, circadian rhythm, chromatin organization, and cell-matrix adhesion (Figure 5B).

Based on high fold regulation, the top activated pathways were proteoglycans in cancer, the Notch signaling pathway, the p53 signaling pathway, endocrine resistance, T helper (Th)1 and Th2 cell differentiation, hematopoietic cell lineage, homologous combinations, and platinum drug resistance (Figure 5C). In addition to this, top activated biological processes were related to cell communication, hemopoiesis, negative regulation of apoptotic processes regulation of cell cycle, DNA replication, angiogenesis, cell proliferation, and DNA repair (Figure 5D).

##### WJT and BMT gliospheres

**Figure 6:**
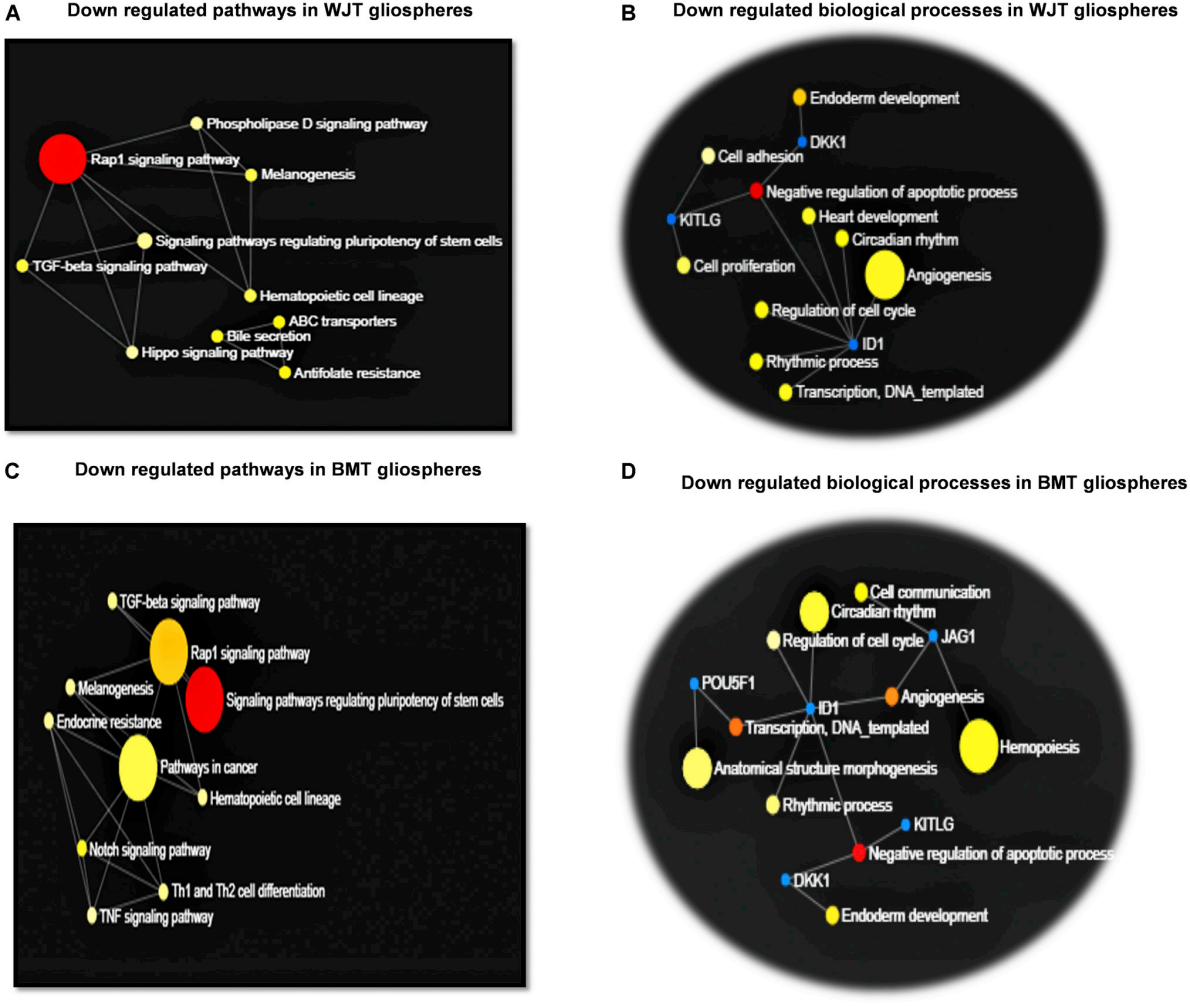
Gene ontology terms of gliospheres treated with CM of WJ-MSCs and BM-MSCs WJT gliospheres. (A) Downregulated KEGG pathways. (B) Downregulated biological processes. BMT gliospheres. (C) Downregulated KEGG pathways. (D) Downregulated biological processes. Both treatments significantly affected the oncogenic activities of GSCs.

Figure 6 shows information about activated and inhibited pathways and biological processes of DEGs of treated gliospheres with two types of CM of MSCs. We found similar activated KEGG pathways (non-significant) from both treatments (Figures S6A and S6B) except complement and coagulant cascade pathways in WJT gliospheres. It was found that both treatments significantly downregulated almost similar pathways with a slight variation (Figure 6). Among downregulated pathways from WJT, the significant ones were *Rap1* signaling, Hippo signaling, *ABC* transporters, signaling pathways regulating pluripotency of stem cells, and transforming growth factor β (*TGF-β*) signaling pathways (Figure 6A). To this, the topmost downregulated biological processes were negative regulation of apoptotic processes, regulation of cell cycle, cell proliferation, and angiogenesis (Figure 6B).

In WJT gliospheres upregulated biological processes related to genes *PLAUR*, *FOXP1*, and *CXCL8* were macrophage activation, inhibition of apoptotic process, heart development, angiogenesis, immune response, and blood coagulation (Figure S6C).

Similarly, Figures 6B and 6D show the downregulated pathways and biological processes of DEGs of BMT gliospheres. It was noted that there was a slight difference in upregulated biological processes for genes *FOXP1*, *CXCL8*, and *NANOG* (Figure S6D). *Nanog* upregulated cell differentiation, cell proliferation, and transcription by RNA polymerase II, while FOXP1 and CXCL8 remained the same as in WJT gliospheres. However, none of these upregulated genes from both treatments was statistically significant.

Alternatively, the significantly downregulated pathways observed from both treatments were signaling pathways regulating pluripotency of stem cells, *Rap 1* signaling, pathways in cancer, *Notch* signaling, and *TGF-β* signaling. Similarly, significantly downregulated biological processes noted were the same, with the highlighted ones being the negative regulation of apoptotic processes, cell communication, angiogenesis, and regulation of cell cycle.

Among upregulated pathways, *AGE-RAGE* signaling and *NF-κB* signaling were the same as shown by microarray analysis as well. This shows that the results of the CSC array are consistent with the findings from the microarray and are being validated. Overall, CSC array analysis depicted the inhibition of cell proliferation, pluripotency, induced differentiation, and activated immune response in GSCs.

#### Significant genes downregulated by both treatments in CSC array

**Figure 7:**
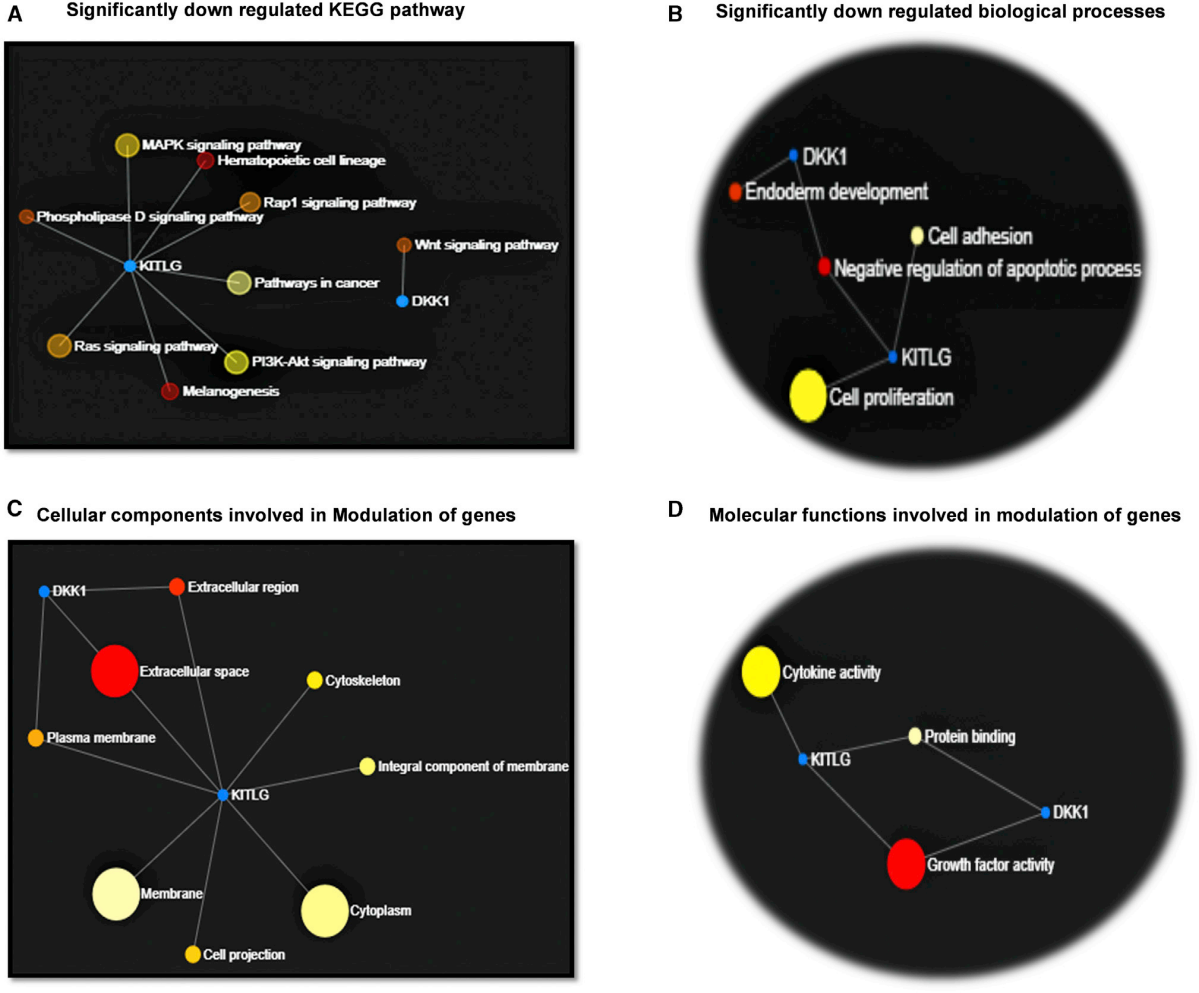
Significant genes affected by both treatments (CM from WJ-MSCs and BM-MSCs) (A) Significantly downregulated genes (p value) and KEGG pathways by both treatments. (B) Significantly downregulated biological processes from two treatments. (C) Significant cellular components activated in response to the effect of two treatments. (D) Significant molecular functions depicting the response of two genes as a result of activation by growth factor and cytokine activity.

It was noted that two genes, *KITLG* and *DKK1*, were significantly downregulated by CM of two types of MSCs. The details of their relevant pathways and biological processes are shown in Figures 7A and 7B. Significantly downregulated pathways found with *KITLG* were hematopoietic cell lineage, melanogenesis, *Rap1 s*ignaling, mitogen-activated protein kinase (*MAPK*) signaling, *P13/AKT* signaling, pathways in cancer, *PLD* signaling, and *Ras* signaling. Alternatively, *DKK1* has been involved in the downregulation of Wnt signaling pathways. About this, the main downregulated biological processes were negative regulation of the apoptotic process, cell proliferation, cell adhesion, and endoderm development. In addition to this, we found that significant cellular components involved to modulate these genes were extracellular region and space, plasma membrane, cytoskeleton, and cellular projections. At the molecular function level, growth factor activity significantly upregulated the DKK1 gene while growth factor and cytokine activity both significantly inhibited the KITLG gene (Figure 7D).

Overall, CM from both MSCs inhibited the cell proliferation, invasion, and pluripotency of GSC mediated by growth factors and cytokines and elicited the immune response that can activate angiogenesis by modulating the tumor microenvironment.

## Discussion

In glioblastoma, GSCs were first identified by Singh et al.^44^ as a population of cells capable of initiating tumor growth *in vivo*. The crucial role played by GSCs in tumor initiation, progression, recurrence, and resistance to therapy indicates that new therapeutic strategies require the eradication of this population.^45^, ^46^, ^47^ Importantly, also note that tumor cells are heterogeneous; therefore, it may be more advantageous to target multiple elements of various cellular pathways to eradicate GBM.^48^ A possible solution to specifically target GSCs might be to force them to acquire a non-self-renewing state. In this non-stem cell-like state, the cells should lose their tumorigenic nature and become vulnerable to therapies. Many therapies fail to have the expected beneficial effects due to the BBB and the presence of active efflux pumps that prevent drug entry into the brain. New treatment modalities, including novel agents and small-molecule inhibitors, are currently under investigation. Remotely, MSCs and their soluble factors are reported to exhibit beneficial anticancer effects.^49^, ^50^, ^51^ To investigate further whether the soluble factors of MSCs may affect different pathways related to proliferation and stemness of GSCs to transform them into non-stem-like cells prone to therapies, we planned this study. An additional objective was to identify whether different kinds of MSCs can exhibit the same anti-tumoral potential at the functional and molecular levels.

In the first step, CD133^+^ cells were sorted and characterized according to morphological and immunocytochemical assays. These cells exhibited a high expression of all of the known markers established for the GSC profile. Actively growing GSCs in spheroids were treated with paracrine factors of manipulated MSCs and showed morphological changes, reduced proliferation, viability, and invasion. The clonogenic assay revealed a remarkable decrease in the self-renewal ability of GSCs, signifying the efficacy of paracrine factors of MSCs. GO results of DEGs from microarray analysis were further evaluated by a specific human CSC array, and we found consistent results of the arrested cell cycle, inhibited metabolism, inhibited pluripotency of GSCs, and activated immune response. IPA analysis of treated spheres indicated inhibited oxidative phosphorylation from both treatments. However, slightly variable canonical pathways were upregulated for immune response from two types of treatments.

Metabolic reprogramming has been the hallmark of CSCs. Growing evidence has demonstrated that slow-cycling GSCs possess a preference for mitochondrial oxidative metabolism. Mitochondrial function plays a crucial role in maintaining stemness and drug resistance of CSCs.^52^, ^53^, ^54^ Few studies have demonstrated that CSCs can rely on fatty acid oxidation for their maintenance and function,^55^ and lipid catabolism seems critical for CSC self-renewal.^56^ Similarly, the mevalonate pathway is an essential metabolic pathway in providing cells with bioactive molecules, crucial for different cellular processes, including cell proliferation, differentiation, survival,^57^ and CSC enrichment.^58^ Since CSCs are enriched in mitochondrial mass and rely heavily on oxidative phosphorylation (OxPhos), disrupting this pathway has become an attractive therapeutic strategy. OxPhos plays a central role in cellular energy. The OxPhos electron transport chain (ETC) constitutes four complexes (CI–CIV) that transfer electrons from donors generated by the tricarboxylic acid (TCA) cycle and fatty acid oxidation to oxygen. Complex V (ATP synthase) uses the stored energy in the proton gradient to generate ATP.^59^, ^60^, ^61^ As shown by our results of IPA (Table 3), the main activated metabolic pathways involved in gliospheres were those with fatty acid and mevalonate pathways that were inhibited due to disruption at C4 and C5 complexes of the ETC in OxPhos (Figure 3C). This may have, in turn, inhibited the proliferation, invasion, and sphere-forming ability of GSCs consistent with the study of Shi et al.^62^

IPA analysis of WJT gliospheres showed activation of *TREM1* signaling, *GP6* signaling, and *NF-κB* signaling. It has been shown that *TREM1* had been upregulated only in infectious inflammatory responses.^63^ In tumors, *TREM1* seems to be induced on tumor-associated macrophages, which has been correlated with cancer recurrence and poor survival.^64^ Immunohistochemical analysis of breast tumor tissues confirmed the co-localization of *TREM1* protein expression with the pan-macrophage marker CD68. These findings established the role of tumor-infiltrating macrophages in promoting inflammation by immune evasion.^65^ It has also been investigated that *TREM1* expression is regulated by *NF-κB* at the transcriptional level,^66^ emphasizing the contribution of *NF-κB* pathway activation in bridging inflammation and tumor promotion and progression.^67^ Hypoxia-regulated genes mediate blood vessel formation by stimulating encoding of chemotactic molecules such as *CCL2*, interleukin-8 (*IL8*), and *VEGF* that recruit macrophages and exert tumor-promoting effects such as angiogenesis.^68^, ^69^, ^70^ Our results are in agreement with the above-mentioned studies given macrophage activation and angiogenesis, as confirmed by the biological processes of treated spheres (Figure 6).

*GPVI* (glycoprotein VI) is exclusively expressed on platelets and megakaryocytes and together with integrin *α2β1* mediates collagen-induced aggregation and adhesion.^71^, ^72^, ^73^ The role of platelets in the pathophysiology of GBM appears to be two-edged. On the one hand, activated platelets and their secretome can modulate immune responses, thereby prolonging overall survival in a GBM model in mice.^74^ On the other hand, platelet activation needs to be avoided since GBM patients have an increased risk for systemic cardiovascular events, and the intratumoral occlusion of numerous vessels leads to a hypoxia-induced tumor progression.^75^ We found that WJT spheres have upregulated a coagulation cascade to recruit platelets as a survival strategy. Therefore, suitable antithrombotic and antiplatelet concepts may be a valuable addition to future individualized, targeted therapies^76^. However, the details of the molecular mechanism in platelet activation require further studies.

IPA analysis of BMT spheres showed upregulation of the *PD-1/PD-L1* pathway and inhibition of calcium-induced T lymphocyte apoptosis. *PD-L1* is not constitutively expressed in tumor cells but rather is inducibly expressed (i.e., adaptive immune resistance) in response to inflammatory signals.^77^^,^^78^ Immune checkpoint inhibitors, *PD-1* and *PD-L1*, have shown clinical efficacies against many different solid and hematologic malignancies.^79^ Binding of *PD-L1* to its receptor suppresses T cell migration, proliferation, and secretion of cytotoxic mediators, and it restricts tumor cell killing. Inhibitors of *PD-1* and *PD-L1* disrupt the PD-1 axis, thereby reversing T cell suppression and enhancing endogenous antitumor immunity to unleash long-term antitumor responses in a wide range of cancers.^80^ Our results show that CM from BM-MSCs have exerted an adaptive immune response, which may have induced *PD-l/PD-L1* expression on cancer cells.

Ca^2+^ signaling plays an essential role throughout vertebrate development, from fertilization to organogenesis. It has been shown that the main checkpoints controlling the fate of a cell are mainly controlled by Ca^2+^ signaling pathways.^81^ A few studies have shown that some tumors develop an immune evasion strategy based on FasL-mediated destruction of invading lymphocytes.^82^, ^83^, ^84^ Invading T lymphocytes that express Fas are stimulated to apoptosis by tumor cells that express FasL. The expression of FasL has recently been demonstrated in GBM.^85^ It has also been reported that T lymphocytes were present in GBM and would account for the aggressive growth of tumors.^86^ Our results show that the calcium-induced T lymphocyte apoptosis pathway has been inhibited, which indicates that CM from BM-MSCs might have affected the Fas-FasL combination and inversed the reaction of immune evasion by GSCs, which in turn upregulated apoptosis in GSCs, as shown by biological process analysis (Figure 6).

From GO results of a specific CSC array, we found two common genes significantly downregulated from CM of both MSCs, such as *KITLG* and *DKK1*.

*DKK1* has downregulated the Wnt pathway (Figure 7A). Many studies have suggested that WNT signaling is aberrantly activated in GBM, and it promotes GBM growth and invasion via the maintenance of stem cell properties.^87^, ^88^, ^89^, ^90^ Dickkopf (*DKK*) acts as an antagonist of WNT signaling via binding to its co-receptor LRP.^91^^,^^92^ Interestingly, the MSC-induced pro-tumorigenic effect seems to be regulated by Wnt/β-catenin signaling in breast cancer,^93^^,^^94^ whereas the inhibition of tumor proliferation occurs by MSC-induced secretion of *DKK-1*, an inhibitor of the same pathway.^95^^,^^96^ Furthermore, the MSC-derived CM exert effects by targeting the *Wnt/β-catenin* signaling pathway.^97^^,^^98^ Our *in silico* results followed these studies, as we found inhibition of growth and stemness properties of GSCs through downregulation of the Wnt pathway mediated by growth factor activity at the molecular level (Figures 7A and 7D).

*KITLG*, also known as the stem cell factor (*SCF*) gene, encodes the ligand of receptor tyrosine kinases (RTKs) by the KIT locus. RTKs are a family of cell surface receptors, which, upon activation, signal through two major downstream pathways, *Ras/MAPK/ERK* and *Ras/PI3K/AKT*. These pathways are involved in the regulation of cell proliferation, survival, differentiation, and angiogenesis.^99^ We found that CM from MSCs inhibited these pathways through the downregulation of the *KITLG* gene mediated by the growth factor and cytokine activity. Also, phospholipase D (*PLD*) activity has been suggested to function as a sensor of metabolites, including lipid pools,^100^ and a critical regulator of autophagy.^101^ Keeping this in mind, we predict that the metabolism of the GSCs was deregulated by inhibition of the *PLD* pathway, which might have upregulated apoptosis and differentiation also shown by biological processes (Figure 7B) mediated by the *KITLG* gene.

Among uniquely upregulated genes of the CSC array of gliospheres, *LIN28B* was found to be the most significant. Lin28, along with Oct4, Sox2, and Nanog, has corroborated its role in pluripotent stem cells.^102^ In addition to tumor initiation, *LIN28B* is necessary for the maintenance of cancers as well.^103^ Recent advances have shown that Lin28 regulates *let-7* miRNA biogenesis and mRNA translation, to coordinate both cellular metabolism and proliferative growth pathways for stem cell self-renewal.^104^ Our results of WJT spheres showed the downregulation of *LIN28B* that might have contributed to the inhibition of metabolism and the self-renewal capacity of GSCs and can alone be considered as a biomarker of GBM stemness after further evaluation at the protein level and using an *in vivo* model.

From BMT spheres, uniquely downregulated genes were *JAG1* and *POU5F1. JAG1* is the ligand of the Notch signaling and has been shown to promote glioma-initiating cells (GICs) in glioblastoma. Notch signaling mediates direct cell-cell interactions and plays a crucial part in cell fate maintenance and self-renewal of GICs.^105^ Moreover, studies have shown that the downregulation of Jagged1 induces apoptosis and inhibits proliferation in glioma cell lines.^106^ Similarly, different variants of *OCT4* (*POU5F1*) have been related to colony formation and regulation of cell survival in GSCs.^107^ We found inhibition of these two vital stem cell markers from CM of BM-MSCs.

One common pathway that has been identified by both arrays is the *AGE-RAGE* signaling pathway. *RAGE* was first identified as a receptor for *AGE* to diabetes, renal diseases, and aging.^108^^,^^109^ In glioblastomas, *RAGE* is expressed on tumor cells, endothelial cells, stromal cells, and tumor-associated macrophages, comprising microglia and myeloid-derived macrophages.^110^ *RAGE* binding activates downstream signaling pathways that stimulate cell proliferation, survival, and migration via increased angiogenesis, inflammation, and reduced apoptosis, while blocking *RAGE* signaling suppresses tumor growth and metastasis.^111^, ^112^, ^113^ Despite the inhibition of oncogenic mechanisms at the cellular level, we found the macrophage activation and immune response as activated biological processes in treated gliospheres. We presume that *AGE-RAGE* signaling might be the contributing factor for this response, which can modulate the tumor microenvironment for angiogenesis. Combining inhibition of *RAGE* signaling while sensitizing CSCs with CM might be a novel strategy to inhibit tumor growth.

There are several challenges involved in treating glioma, including the immunosuppressive nature of GBM itself with high inhibitory checkpoint expression, the immunoselection BBB impairing the ability for peripheral lymphocytes to traffic to the tumor microenvironment, and the high prevalence of corticosteroid use, all of which suppress lymphocyte activation. However, by simultaneously targeting multiple costimulatory and inhibitory pathways, it may be possible to achieve an effective antitumoral immune response.^114^ This is where a combination of manipulated MSC-secreted factors has the most significant potential for cell-free-based anticancer therapies.

### Conclusions

Taken together, the results of microarray and CSC arrays, *in vitro*, elucidate the possible molecular targets by which secreted factors of MSCs inhibited the 3D formation of GSCs observed in culture. The inhibition was translated into decreased oncogenic activities, including stemness of GSCs through different pathways mediated by *KITLG* and *DKK1* genes. We conclude that growth factors and cytokines in CM from two sources of MSCs hold the antitumor properties, which are mediated by different routes of signaling pathways while causing the same effect. It has been shown that neurotrophic factors in CM could access affected neurons in the central nervous system (CNS) by either directly crossing the BBB or through the retrograde transport mechanism in the CNS. CM have already been implicated for many neurodegenerative diseases,^115^, ^116^, ^117^ and therefore CM from MSCs can be a subject of combinatorial therapy for gliomas. Regarding the preference of choice, we propose that CM from BM-MSCs may contribute a more valuable effect as a combinatorial therapy in conjunction with antitumor immune therapy to treat gliomas since it has inhibited the T lymphocyte apoptosis pathway to facilitate the immune reaction and also inhibited the pluripotent stem cell markers. This study has provided valuable information regarding the potential ability of acclimatized MSCs with the GSC environment to interrupt the growth and pluripotency of GSCs with the potential for translation to practical treatment options for GBM in human patients. Furthermore, *in vivo* studies along with proteomics are warranted to translate the apparent therapeutic efficacy of CM of MSCs in the treatment of glioblastoma. Moreover, identification of the specific growth factors and cytokines responsible for the inhibitory response will provide useful information for developing an effective paradigm for glioblastoma treatment.

## Materials and methods

### Isolation of GSCs from GBM cell line U87MG

#### Monolayer generation of cell line

The GBM cell line U87MG (ATCC HTB14TM) was expanded according to the protocol described by ATCC with a slight modification. Briefly, the cell line was expanded in standard culture media (DMEM-F12 1:1, l-glutamine 200 mM, 10,000 U/mL penicillin/streptomycin, 25 μg/mL amphotericin B, and 10% heat-inactivated fetal bovine serum [FBS]; Gibco/Invitrogen, USA). The cells were seeded at 1 × 10^4^cells/cm^2^ in T75 cultures flasks and maintained at 37°C and 5% CO_2_. The culture medium was exchanged every 2–3 days.

#### FACS sorting and enrichment of glioma stem cells

The cell line (U87MG) expanded at different passages (P3–P9) was investigated for the percentage of the CD133^+^ population, which was sorted by using a fluorescence-activated cell sorting (FACS) JAZZ cell sorter (BD Biosciences, USA) according to the manufacturer’s instruction. Briefly, upon reaching 80%–90% confluence, U87MG cells were harvested using 0.25% trypsin EDTA (Invitrogen, USA), washed twice with cold PBS (Invitrogen, USA), and centrifuged at 300 × *g* for 5 min. The cell pellet was re-suspended in 100 μL of BD FACS staining buffer (BD Biosciences, USA), and 10 μL of CD133 antibody (eBioscience, USA) was added, mixed well, and put at 4°C for about 30 min in the dark. Next, 500 μL of cold PBS was added to wash the cells and centrifuged at 300 × *g* for 5 min, and the pellet was re-suspended in 500 μL of PBS for sorting. Sorted populations were divided into positive and negative fractions and were evaluated for the enrichment of CD133^+^ cells using a FACSCanto II and analyzed by FACSDiva software version 7 (BD Biosciences, USA).

### Characterization of sorted populations

#### First gliosphere generation

Sorted tumor cells from both positive and negative populations were directly seeded in CSC media (termed herein as GSM) (DMEM-F12 [Gibco, USA] supplemented with N2 [Gibco, USA], epidermal growth factor [EGF] [20 ng/mL, Invitrogen], basic fibroblast growth factor [bFGF] [20 ng/mL, Gibco, USA], leukemia inhibitory factor [LIF] [10 ng/μL, Chemicon, Germany], and B27 [1:50; Life Technologies, USA] in six-well ultralow attachment plates (Corning, USA) to enrich for glioma stem cells (gliospheres) and were maintained at 37°C in a humidified atmosphere of 5% CO_2_/95% air. The morphology of gliospheres from positive and negative populations was observed under a phase-contrast microscope (Zeiss, Germany) for 10 days.

#### Glio-sub-sphere generation

CD133^+^ and CD133^−^ selected cell populations, derived from first gliospheres, were dissociated using StemPro Accutase cell dissociation reagent (Gibco, USA) according to the manufacturer’s instructions. Cells were subsequently re-suspended in GSM and seeded in 24-well ultralow plates (Corning, USA) at 1 × 10^3^ cells/well. The formation of free-floating sub-spheres was observed by phase-contrast microscopy (Zeiss, Germany), and the experiment was conducted until passage 12 (G12, gliosphere). Cells in each passage were kept in culture between 7 and 10 days.

#### Monolayer generation

Similarly, small fractions of sorted cells from positive and negative populations were cultured in standard culture media (DMEM-F12 + 10% FBS, Gibco, USA) to investigate their expansion ability adherently after sorting. The morphological changes were observed under a phase-contrast microscope (Zeiss, Germany). The experiment was conducted for six passages, and one passage time was kept between 5 and 7 days.

#### Immunofluorescence assay of CD133^+^ population

A panel of GSC markers, neuronal lineage markers, and late differentiated neuronal markers were selected (Table 1) to characterize the sorted CD133^+^ population by immunocytochemistry. Briefly, sub-spheres at passage 13 (G13) were harvested by treatment with StemPro Accutase cell dissociation reagent (Gibco, USA) and washed with PBS (pH 7.4, Gibco, USA), followed by centrifugation. After resuspension, cells were seeded on poly-l-lysine-coated coverslips at a seeding density of 2 × 10^4^ cells/coverslip and were immersed in GSM with 2% FBS (Gibco, USA). After 2 days the cells were fixed with 4% formaldehyde for 20 min followed by washing. Coverslips were then permeabilized with 0.3% Triton X-100 for 15 min at room temperature. Blocking was performed with 10% normal goat serum (Gibco, USA) for 1 h at room temperature. Cells were stained with the primary antibodies in 1% blocking buffer overnight at 40°C with shaking followed by washing with 0.1% Triton X-100 in PBS three times for 5 min each. Next, cells were stained with secondary antibodies (Table 1) in 1% blocking buffer for 1 h at room temperature in the dark. Cells were washed with 0.1% Triton X-100 in PBS three times for 5 min each. Nuclei were stained with DAPI (Thermo Fisher Scientific, USA) for 5 min and coverslips were mounted onto slides. Cell imaging was performed on an inverted phase-contrast fluorescence microscope (Zeiss Axio Observer Z1, Zeiss, Germany). The description of the primary and secondary antibodies used for the assay is given in Table 1.

### Functional assay*s*

#### Preparation of MSC CM under GSM conditions

BM-MSCs and WJ-MSCs were acquired from the Cell Therapy Center, The University of Jordan, which had been expanded and characterized as described previously.^39^ Three biological samples of WJ-MSCs (WJ1, WJ2, WJ3) and three from BM-MSCs (BM1, BM2, BM3) at G3 were used to generate CM. All six cell lines were plated into T75 flasks (TPP, USA) at a density of 5 × 10^3^ cells/cm^2^ in alpha-minimum essential medium (MEM) (Gibco, USA) + 5% platelet lysate.^40^ A day before cells reached 80% confluency, monolayers were washed twice with PBS (pH 7.4) and once with SFM (DMEM-F12). Next, GSM and SFM were added in the flasks and after 48 h the CM (CM as GSCM and SFCM) were collected, centrifuged at 300 × *g* for 10 min at 4°C, filtered through 0.2-μm filters (BD Biosciences, USA), and aliquoted. The CM were stored at −80°C and fresh aliquots were used before each experimental assay. All experiments were performed from CM of individual biological samples from two types of MSCs; however, for simplicity we have used the general terms of BM-MSCs and WJ-MSCs herein.

#### Optimization of CM concentrations

Initially different concentrations of CM were tested on morphology and proliferation potential of glioma stem cells (Figures S1A and S1B). As described above, GSCM and SFCM were tested as 100% and 50% with fresh GSM, from one sample of BM1-MSCs and WJ3-MSCs, and SFM and GSM were kept as controls. These experiments were conducted for 4 days (96 h) only. Based on these results, further experiments were planned from 100% GSCM for 7 days, and to replenish the growth factors in the GSCM, we used the CM in a ratio of 10%:90% (GSM/GSCM). For simplicity, we have used the term CM herein.

### Assessment of the effects of CM on GSCs (gliospheres)

#### Morphological assessment

Briefly, 1 × 10^3^ cells/cm^2^ from dissociated gliospheres at G13 were plated in ultralow attachment six-well plates (Corning, USA) containing CM. The experiment was conducted for 7 days, and cells growing in normal GSM were kept as a control. CM was added every 48 h, and changes in the sphere-forming ability of GSCs were captured by phase-contrast microscopy (Zeiss, Germany).

#### 3D cell proliferation and viability assessment

The proliferation rate of GSCs (at G13) was evaluated by a CellTiter-Glo 3D cell viability assay (Promega, USA). In brief, the cells were cultured at a seeding density of 5 × 10^3^ cells/well in 100 μL of CM in 96-well ultralow attachment plates (Corning, USA). Every 48 h fresh CM were added along with control and the experiment was conducted for 7 days. The proliferation rate was determined at days 3 and 7 by CellTiter-Glo 3D cell viability reagent according to the manufacturer’s instruction (Promega, USA). Luminescence was measured using a microplate reader (GloMax-multi detection system, Promega, USA).

#### Invasion assay

To determine the effect of CM on the invasive ability of GSCs, an invasion assay was performed using a Cultrex BME cell invasion assay (Trevigen, USA) with a slight modification. Briefly, 5 × 10^4^ cells were treated with CM from both types of MSCs in six-well ultralow attachment plates (Corning, USA) for 7 days. The media were refreshed every 48 h. On day 7 the spheres were collected and harvested by Accutase (Gibco, USA), centrifuged at 200 × *g* for 4 min, and 5 × 10^4^ cells were seeded in the upper chamber of the BME 96-well plate. The lower chamber was filled with serum media (DMEM-F12 + 10% FBS, Gibco, USA). After 2 days the plates were processed according to the manufacturer’s instruction and the luminescence was measured using a microplate reader (GloMax-multi detection system, Promega, USA).

#### Clonogenic assay

To investigate whether the effect exerted by CM from both types of MSCs is reversible or irreversible, clonogenic assays (colony-forming efficiency [CFE] and sphere-forming efficiency [SFE]) were performed for both adherent and spheroid system.

##### CFE

Briefly, 5 × 10^3^ cells/well were treated in 24-well ultralow plates (Corning, USA) with CM from both types of MSCs for 7 days along with control. After harvesting, single-cell suspensions were obtained. 50 cells from each sample were seeded in six-well plates (TPP, USA) in standard culture media (DMEM-F12 + 10% FBS) for 2 weeks (14 days). The medium was exchanged every 3 days. Colonies were stained using 0.5% crystal violet dye (Sigma-Aldrich, USA) according to the manufacturer’s instructions and counted by a light microscope (Zeiss, Germany).

##### SFE

Similarly, after dissociation of treated spheres, 2 × 10^3^ cells/well were cultured in 24-well ultralow plates (Corning, USA) in GSM for about 7 days. GSM were added every 48 h and sphere formation was monitored under a light microscope (Zeiss, Germany). On day 8 the spheres were counted and imaged using a Zeiss microscope. Spheres were dissociated again as described before and all cells were plated for successive passage. Fresh GSM were added every 48 h, and passage time was kept for 7 days.

### Molecular assays (microarrays and CSC arrays)

#### RNA extraction

Briefly, 1 × 10^4^ cells/cm^2^ (G13) were seeded in 25 mL of ultralow attachment flasks (Corning, USA) containing CM from two types of MSCs and control (gliospheres). The sorted population that did not make spheres was taken as a negative control. On day 7 the spheres were harvested using Accutase (Gibco, USA). Total RNA was extracted using TRIzol reagent (Invitrogen, CA, USA) and cleaned up with the RNeasy mini kit (QIAGEN, USA) following the instructions of the manufacturer. Extracted RNA was quantified using the NanoDrop 2000c spectrophotometer system (Thermo Fisher Scientific, USA). RNA quality analysis was performed using an Agilent 2100 Bioanalyzer instrument with an Agilent RNA 6000 Nano kit according to the manufacturer’s instructions.

#### Global gene expression profiling (transcriptome analysis)

Whole transcriptome analysis was performed in triplicate for gliospheres, control group (−Ve and cell line), gliospheres treated with CM from BM-MSCs (BMT), and CM from WJ-MSCs (WJT). GeneChip Human Transcriptome Array (HTA) 2.0 (Affymetrix, Santa Clara, CA, USA) was used for gene expression profile analysis. The procedure was followed as described by the manufacturer. The microarray data can be accessed via Gene Expression Omnibus (GEO: GSE149216). For simplicity, we use the terms BMT and WJT for treated gliospheres herein.

#### Data analysis

Raw CEL file normalization was performed using the signal space transformation-robust multi-array analysis (SST-RMA) using Affymetrix Expression Console software (transcription analysis console [TAC]) version 4.0.1 (Affymetrix). DEGs at FC (log_2_) of ≥2 or –2 or less with a statistical significance level of p <0.05 (gliospheres versus BMT gliospheres, gliospheres versus WJT gliospheres, and gliospheres versus control) were picked up. GO term and pathway enrichment analyses were conducted to determine the roles of these DEGs by IPA software (Ingenuity Systems, Redwood City, CA, USA), NetworkAnalyst (3.0) (https://www.networkanalyst.ca/), and using the open-source Database for Annotation, Visualization and Integrated Discovery (DAVID) v6.8 (https://david.ncifcrf.gov/home.jsp).

#### RT^2^ Profiler PCR array

To validate further the specific genes and pathways involved in certain mechanisms exhibited by transcriptomic analysis and particularly the effect on stemness, CSC arrays were performed and analyzed according to the manufacturer’s instructions (QIAGEN, USA). Briefly, cDNA was synthesized using the RT^2^ First Strand Kit (catalog no. 330404, QIAGEN, USA). A diluted cDNA aliquot was mixed with RT2 SYBR Green qPCR mastermix (catalog no. 330503, QIAGEN, USA) and loaded into the 96-well array plate of RT2 Profiler PCR array human CSCs (catalog no. PAHS-176Z, QIAGEN, USA). qPCR reactions were performed using the CFX96 C1000 Touch thermal cycler (Bio-Rad, USA) with the following temperature setting: (1) 95°C for 10 min, and (2) 40 cycles of 95°C for 15 s and 60°C for 1 min. The data analysis was performed using the 2^−ΔΔCt^ method available by the SABiosciences company (QIAGEN, USA). The data were normalized, across all plates, to the average of the arithmetic mean of the following housekeeping genes: glyceraldehyde 3-phosphate dehydrogenase (GAPDH), β_2_-microglobulin (B2M), and actin beta (ACTB). The threshold cycle values of the control wells were all within the ranges recommended by the PCR array user manual.

### Statistical analysis

All experiments were performed in triplicates (n = 3). Data were analyzed using Microsoft Excel and GraphPad Prism software. Quantitative data were expressed as mean ± standard deviation. Data were evaluated by two-way ANOVA, and Dunnett’s post-test was used to analyze multiple comparisons (p < 0.05). All procedures performed in this study were in accordance with the instructions from institutional review board of the Cell Therapy Center.

## Supplemental information

Document S1. Figures S1–S7

Supplementary file (Excel 1)

Document S2. Article plus supplemental information
