# Supplementary material for: Anti-oncogenic activities exhibited by paracrine factors of MSCs can be mediated by modulation of KITLG and DKK1 genes in glioma SCs in vitro
Source: Mol Ther Oncolytics. 2020 Nov 26;20:147–65. doi: 10.1016/j.omto.2020.11.005 (PMC7851499; doi:10.1016/j.omto.2020.11.005)
Supplement: Document S1. Figures S1–S7 [file mmc1.pdf]

OMTO, Volume *20*

Supplemental Information

Anti-oncogenic activities exhibited by paracrine factors of MSCs can be mediated by modulation of *KITLG* and *DKK1* genes in glioma SCs *in vitro*

Nazneen Aslam, Elham Abusharieh, Duaa Abuarqoub, Dema Ali, Dana Al-Hattab, Suha Wehaibi, Ban Al-Kurdi, Fatima Jamali, Walhan Alshaer, Hanan Jafar, and Abdalla S. Awidi

Figure. S1

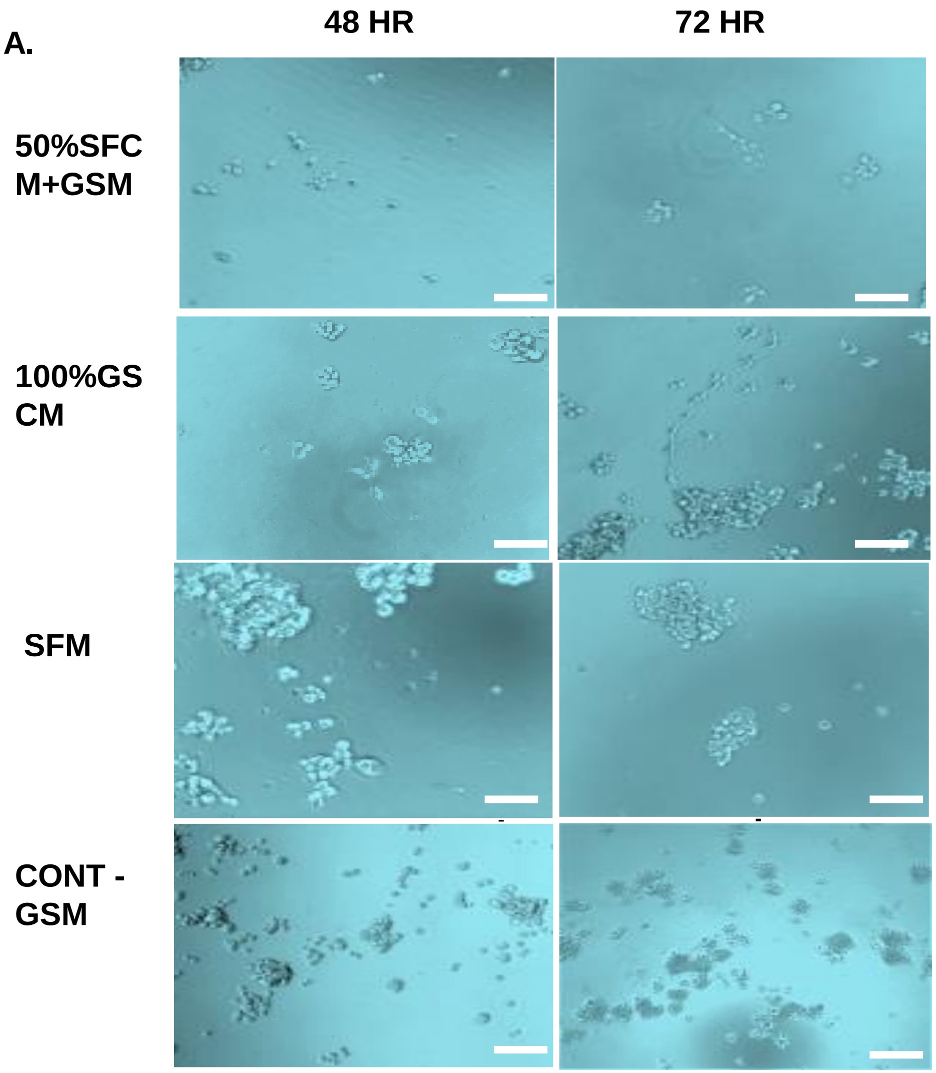

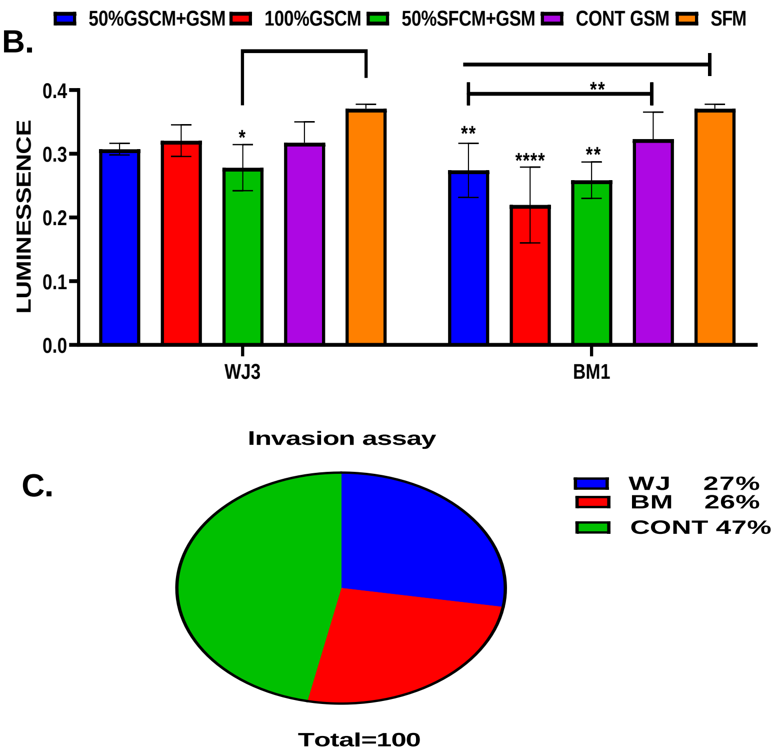

D. Clonogenic assay

	Spheres(3D)	Colonies(2D)
WJ-MSCs	7.0	6.0
BM-MSCs	5.0	11
Control	39	19

Figure.S1. Preliminary data, Invasion assay and Clonogenic assay.
A. Representative images of the effect of different concentrations of CM (SFCM and GSCM) on the morphology of spheroids in comparison to both controls at 48HR and 72HR. **B.** Effect of different concentrations of CM (SFCM and GSCM) on the proliferation of gliospheres from one sample of BM1-MSCs and WJ3-MSCs for 96HR ($p<0.05, 0.01, 0.0001$). **C.** Invasion assay showing percentage of GSCs inhibited after treatment with CM from two types of MSCs. **D.** Clonogenic assay after treatment with CM from two types of MSCs.

Figure. S2

A. Percentages of CD133 population before and after sorting

B. After sorting enrichment in sphere culture

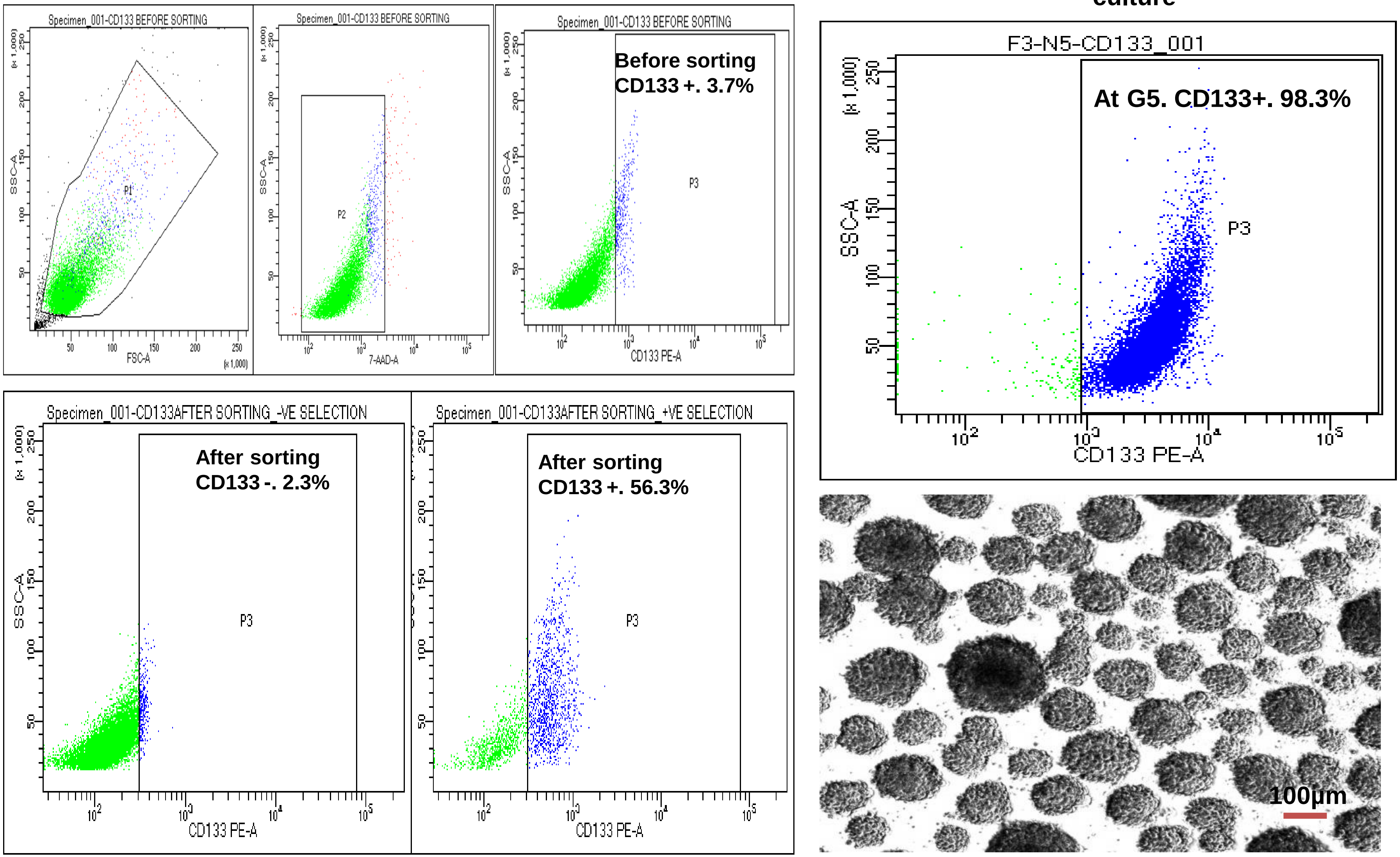

Figure.S2. Flow Cytometric evaluation of CD133 populations.

A. Flow cytometric evaluation of the percentages of CD133 population before sorting and enrichment soon after sorting. **B.** Enrichment of CD133+ population in sphere culture at G5 with representative image of the spheres morphology. Scale bar.100µm.

Figure.S3

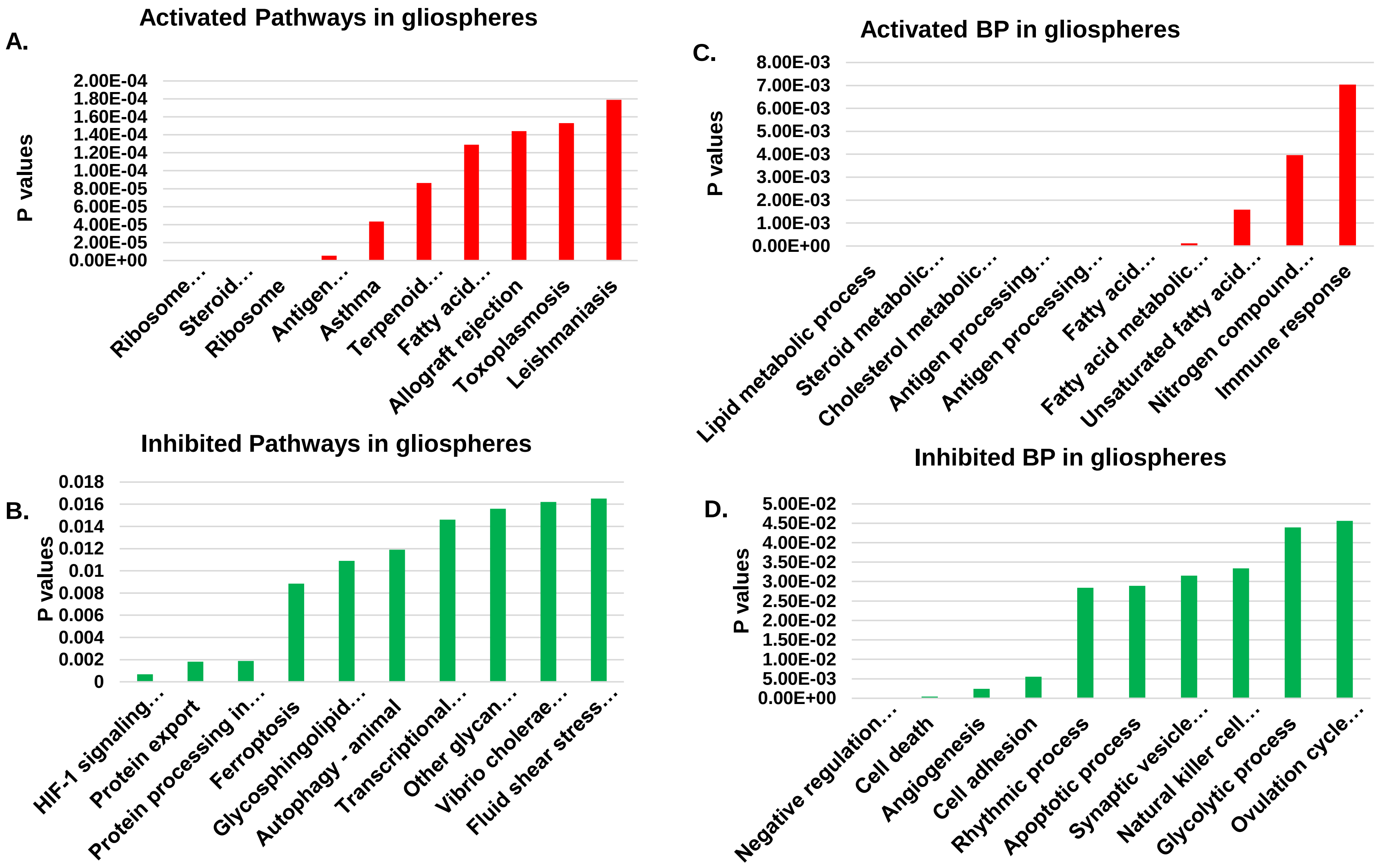

Figure.S3. Gene ontology terms of gliospheres vs control group (+VeS vs Cell line and –Ve) from microarray result. **A.** Activated KEGG pathways**,** **B.** Inhibited KEGG pathways, **C.** Activated biological processes, **D.** Inhibited biological processes.

Figure. S4

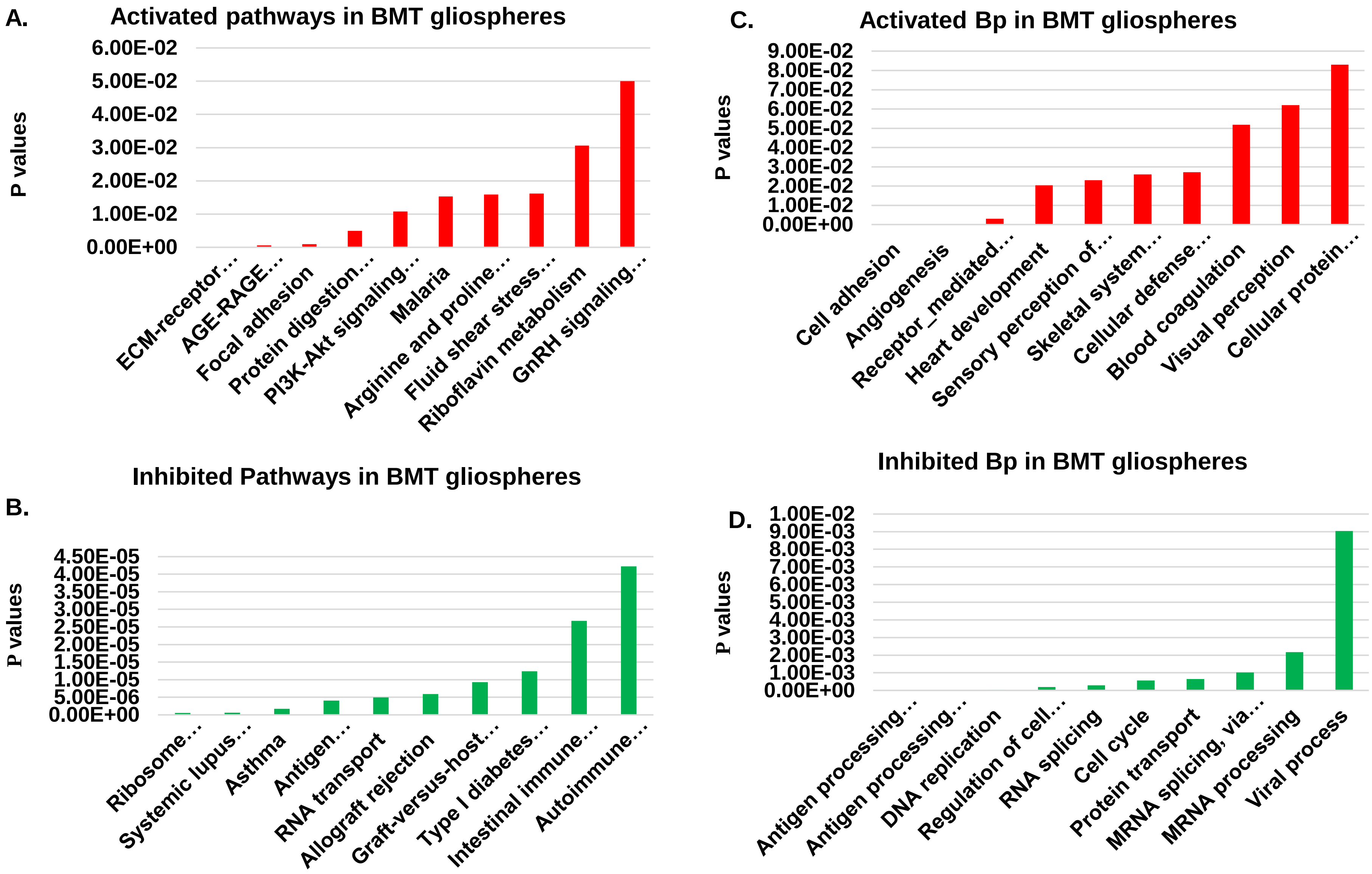

Figure.S4. Gene ontology terms of treated gliospheres with CM-BMMSCs vs gliospheres (BMT vs +VeS)
A. Activated KEGG pathways, **B**. Inhibited KEGG pathways, **C.** Activated biological processes, **D.** Inhibited biological processes.

Figure. S5

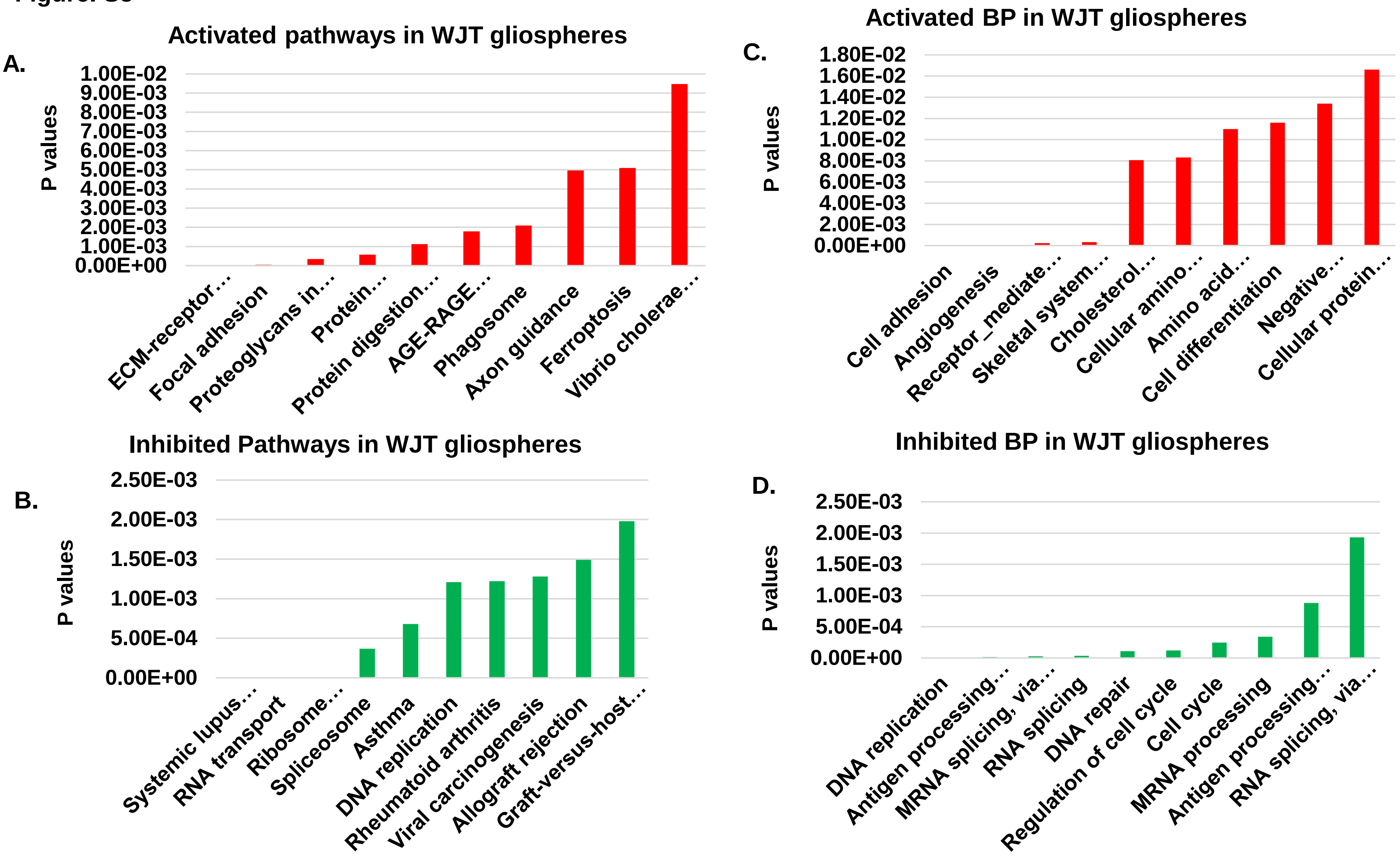

Figure.S5. Gene ontology terms of treated gliospheres with CM-WJMSCs vs gliospheres (WJT vs +VeS). **A.** Activated KEGG pathways, **B**. Inhibited KEGG pathways, **C**. Activated biological processes, **D.** Inhibited biological processes.

Figure.S6

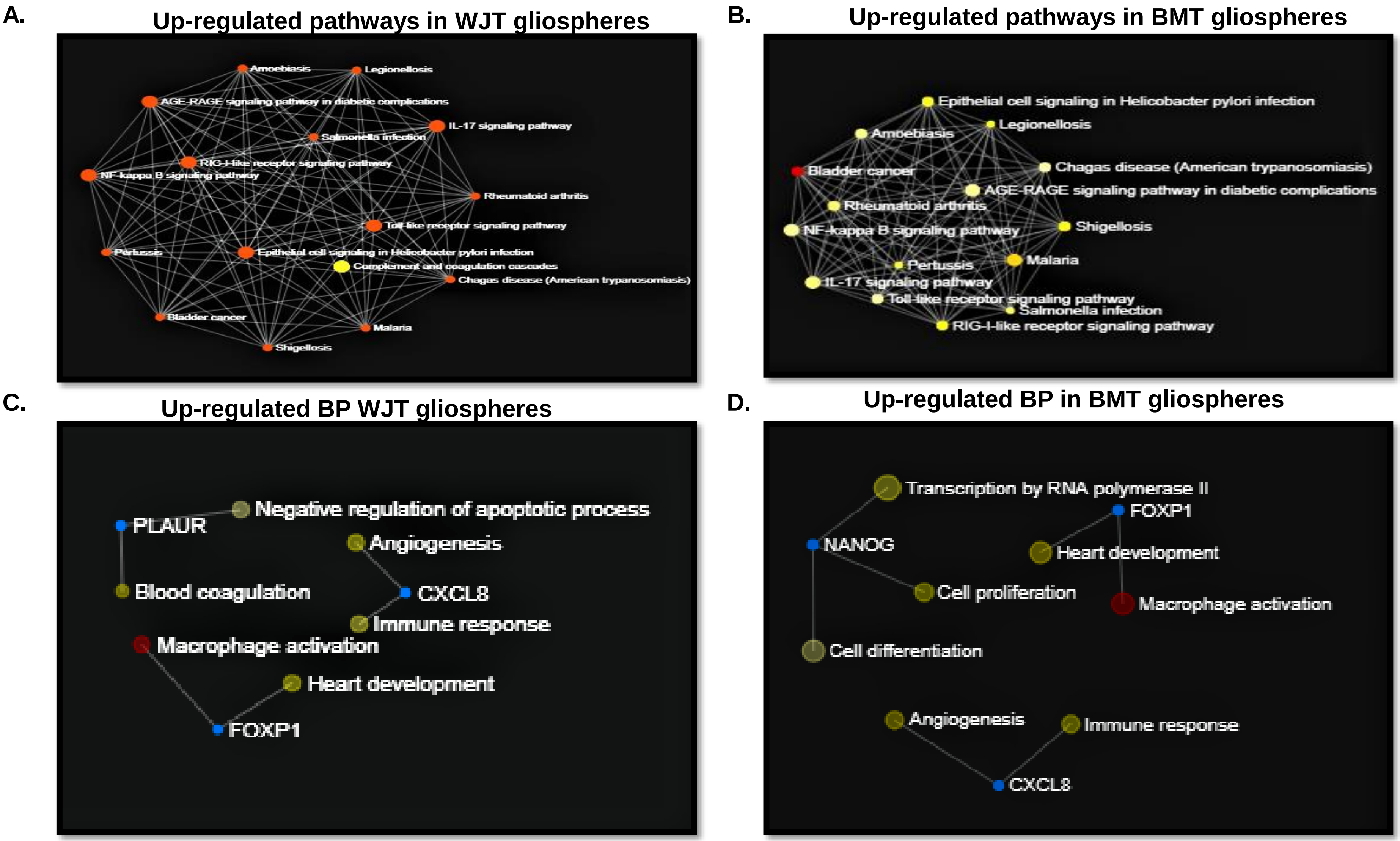

Figure. S6. Gene ontology of treated gliospheres from CSC array results.
A. Up-regulated KEGG pathways in WJT gliospheres, **B**. Up-regulated KEGG pathways in BMT gliospheres, **C**. Up-regulated biological processes (WJT), **D**. Up-regulated biological processes (BMT).

Figure.S7

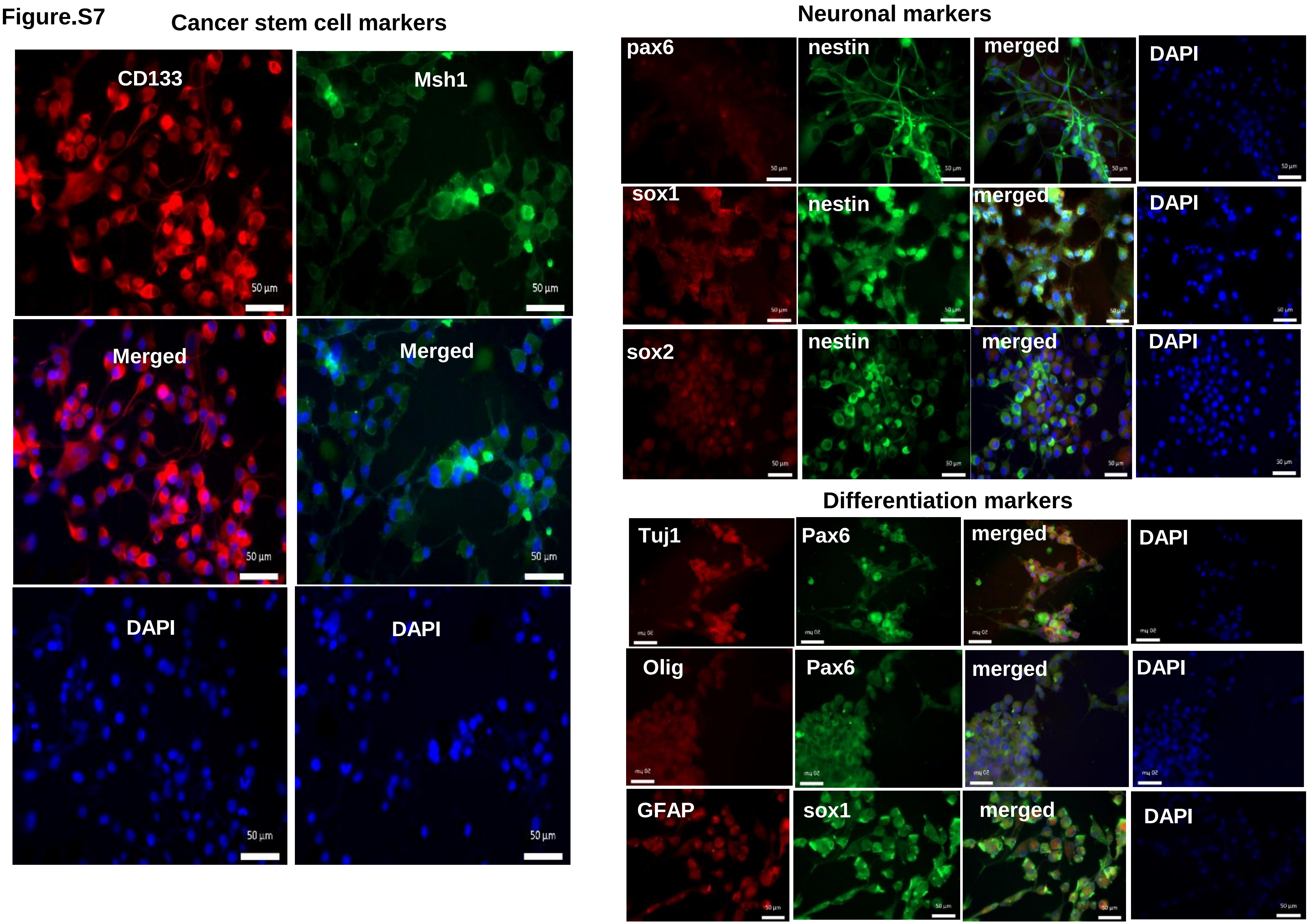

Figure. S7. Immunocytochemical assay for characterization of GSCs.
Immunocytochemical assay showing the panels of markers used for characterization of glioma stem cells (See Figure 1C).
